# The Lewis Pair Polymerization of Lactones Using Metal Halides and *N*-Heterocyclic Olefins: Theoretical Insights

**DOI:** 10.3390/molecules23020432

**Published:** 2018-02-15

**Authors:** Jan Meisner, Johannes Karwounopoulos, Patrick Walther, Johannes Kästner, Stefan Naumann

**Affiliations:** 1 Institute of Theoretical Chemistry, University of Stuttgart, Pfaffenwaldring 55, D-70569 Stuttgart, Germany; meisner@theochem.uni-stuttgart.de (J.M.); johannes.karwou@googlemail.com (J.K.); kaestner@theochem.uni-stuttgart.de (J.K.); 2 Institute of Polymer Chemistry, University of Stuttgart, Pfaffenwaldring 55, D-70569 Stuttgart, Germany; patrick.walther@ipoc.uni-stuttgart.de

**Keywords:** Lewis pairs, polyesters, *N*-heterocyclic olefins, density functional theory

## Abstract

Lewis pair polymerization employing *N*-Heterocyclic olefins (NHOs) and simple metal halides as co-catalysts has emerged as a useful tool to polymerize diverse lactones. To elucidate some of the mechanistic aspects that remain unclear to date and to better understand the impact of the metal species, computational methods have been applied. Several key aspects have been considered: (1) the formation of NHO-metal halide adducts has been evaluated for eight different NHOs and three different Lewis acids, (2) the coordination of four lactones to MgCl_2_ was studied and (3) the deprotonation of an initiator (butanol) was investigated in the presence and absence of metal halide for one specific Lewis pair. It was found that the propensity for adduct formation can be influenced, perhaps even designed, by varying both organic and metallic components. Apart from the NHO backbone, the substituents on the exocyclic, olefinic carbon have emerged as interesting tuning site. The tendency to form adducts is ZnCl_2_ > MgCl_2_ > LiCl. If lactones coordinate to MgCl_2_, the most likely binding mode is via the carbonyl oxygen. A chelating coordination cannot be ruled out and seems to gain importance upon increasing ring-size of the lactone. For a representative NHO, it is demonstrated that in a metal-free setting an initiating alcohol cannot be deprotonated, while in the presence of MgCl_2_ the same process is exothermic with a low barrier.

## 1. Introduction

**Scheme 1:**
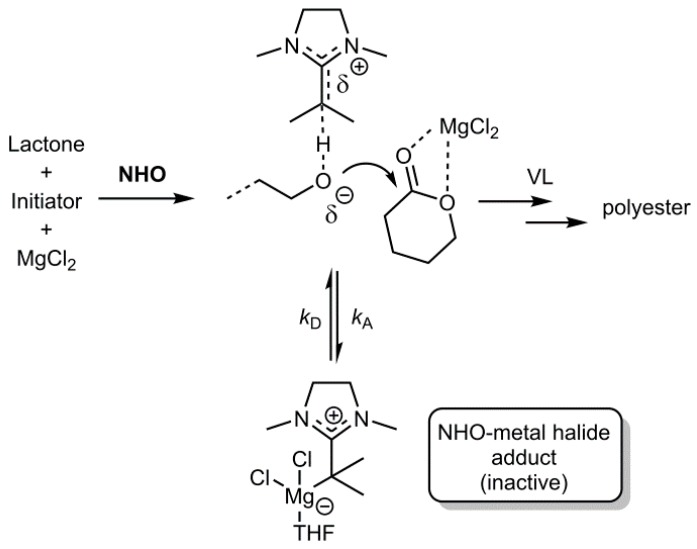
Simplified reaction mechanism for the cooperative activation of initiators/propagating chain ends and monomers in the *N*-Heterocyclic olefin (NHO)/metal halide Lewis pair (LP)-catalyzed polymerization of lactones. The adduct is assumed to be inactive.

**Figure 1:**
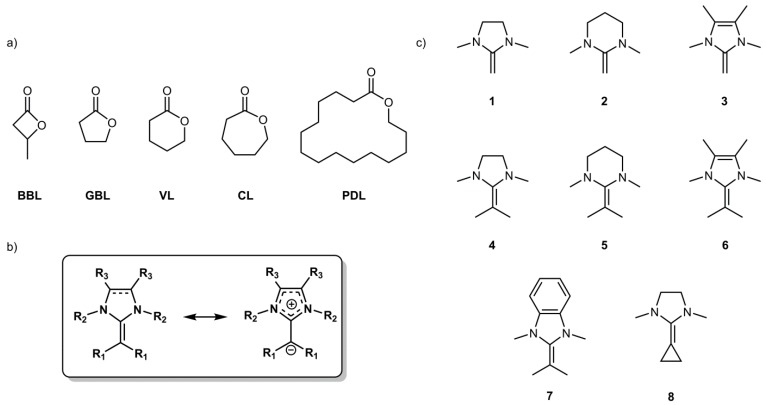
(**a**) Different lactones that have been polymerized by NHO/metal halide LPs; (**b**) mesomeric structure emphasizing the polarized character of the NHO double bond; (**c**) range of NHOs considered in this work. BBL: β-butyrolactone; GBL: γ-butyrolactone; VL: *δ*-valerolactone; CL: *ε*-caprolactone; PDL: pentadecalactone.

Among the many approaches towards polymerization-active Lewis pairs (LPs) [1,2,3,4,5,6,7,8,9], the combination of commercially available metal halides (MX_n_) and readily accessible organobase catalysts (OBCs) provides very simple, yet surprisingly powerful access to LP catalysts for lactone polymerization [10,11,12]. Thereby, it is assumed that the Lewis acid (MgCl_2_, ZnCl_2_, LiCl…) coordinates to the monomer, accepting electron density and thus increases the polarization of the carbonyl group (Scheme 1). This facilitates attack by an (organic) nucleophile (i.e., an alcohol initiator activated by the applied OBC) and subsequent ring-opening. This has repeatedly been described to result in increased polymerization rates, including cases where it is only the cooperative interaction between Lewis base and Lewis acid which engenders reactivity in the catalytic setup—quite frequently, the individual components are fully polymerization-inactive on their own [2,4,10,11,12]. Our group has recently expanded on several other key aspects of this chemistry [10,11,12]. For one, it was demonstrated that reactivity and selectivity can be decoupled for many examples of MX_n_/OBC Lewis pair polymerization of lactones. This means that higher polymerization rates are not simultaneously accompanied by increased transesterification, the major side reaction responsible for broad molecular weight distributions and limited molecular weights. This beneficial behavior was attributed to the ability of the activating Lewis acid to differentiate between the polymer chain and the cyclic monomer. Moreover, the inherent adaptability of this setup (both components can be exchanged independently) has enabled a broad applicability: lactone monomers as different as γ-butyrolactone (GBL, Figure 1), δ-valerolactone (VL), ε-caprolactone (CL) or pentadecalactone (PDL) were successfully homo- or copolymerized. Crucially, the choice of activating Lewis acid not only modulates the polymerization rates, but is also a convenient tool to manipulate the copolymerization parameters. The preparation of aliphatic polyester with different compositions from the same 1:1 comonomer feed can thus be realized; the incorporation of significant amounts of GBL was also possible [11,12]. This effect was proposed to originate from a certain degree of monomer selectivity displayed by the activating Lewis acid. Different metal halides will activate different lactone monomers to a varying extent because they differ in parameters such as steric hindrance, ion radii, monomer-metal complex stability or the O–C–O angle of the lactone.

During these studies, *N*-heterocyclic olefins (NHOs) have been identified as especially suited for these applications. NHOs are reactive OBCs on account of their highly polarized double bond (Figure 1) [13,14,15,16,17,18]; the high electron density on the exocyclic carbon renders it nucleophilic [19] and a strong Brønsted-base [20]. Additionally, this polarization can be readily tuned and synthesis is usually limited to few and simple steps [21,22,23]. Owing to their steadily increasing popularity, NHOs and their structural and catalytic properties have also been the focus of other detailed theoretical studies [24,25,26,27,28,29,30,31].

**Figure 2:**
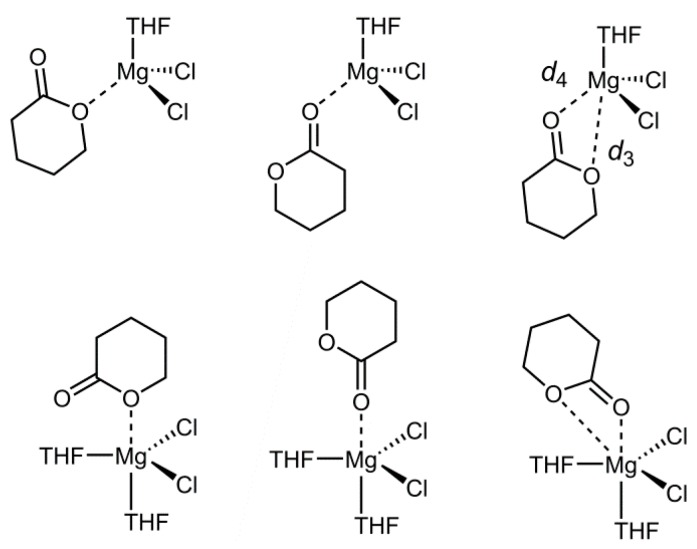
Investigated types of lactone-ligated magnesium complexes. Top: Tetrahedral geometry; Bottom: Trigonal-bipyramidal coordination. From left to right: endocyclic activation, carbonyl activation and chelating activation.

In view of the high performance and promising prospects of this type of Lewis pair polymerization, a better understanding of the underlying mechanisms is much desired. A rewarding long-term goal would be the ability to design the MX_n_/OBC pairs so as to tailor the resulting polymer material with regard to architecture (random, gradient, block-like) and composition (comonomer content), arriving at a polyester with rationally pre-determined properties. However, presently, systematic investigations on this subject are missing and even very basic questions remain unresolved. For example, it is not clear how the Lewis acid actually coordinates with the lactone; three different modes can be envisioned (Figure 2). The influence of the Lewis acid on initiation and propagation is equally diffuse as yet, and coherently the origin of the experimentally observed monomer selectivity is still elusive.

While this work does not attempt to describe a comprehensive mechanistic scenario for ring-opening polymerization using NHO/metal halide LPs, it is intended to elucidate some central aspects of the discussed polymerization pathways by way of density functional theory (DFT) calculations. We have included several different metal halides in this study (MgCl_2_, ZnCl_2_, LiCl), as well as a representative number of various NHO structures (**1**–**8**, Figure 1) and lactone monomers. The issues investigated in the following are (i) the stability of NHO/MX_n_ adducts; (ii) the lactone/MX_n_ coordination (mode) and (iii) finally the proton transfer from the initiator to the NHO in the presence of a Lewis acid, detailed for one example. 

## 2. Results

### 2.1. Lewis Acid—Lewis Base Adducts

**Scheme 2:**
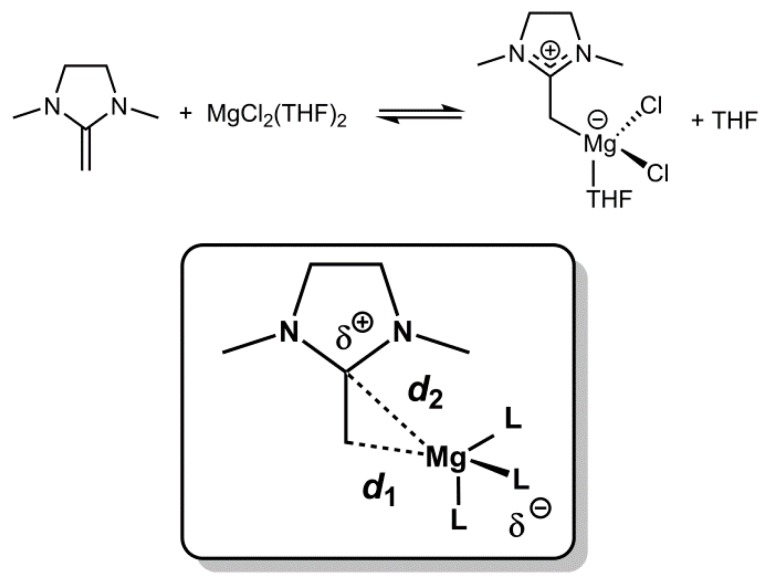
Investigated formation of NHO/Lewis acid adducts and definition of *d*_1_/*d*_2_.

As a first step, NHO-adduct formation was investigated. Since the currently proposed polymerization mechanisms [11,12] are based on the assumption of a free NHO acting in cooperation with a free, i.e., non-complexed, Lewis acid, a sufficient lability of potential NHO-metal complexes in a given LP polymerization system must be ensured. The formation of too stable adducts would quench any polymerization activity; on the other hand, the choice of a too weakly coordinating metal ion will also impair its ability to activate lactones for ring-opening—an issue that has been identified as central in several polymerization systems employing *N*-heterocyclic carbenes (NHCs) or NHOs [4,10,11,12,32]. Thus, in order to achieve a better understanding of this required balance, the affinity of eight structurally different NHOs (**1**–**8**, Figure 1) to bind to each of the Lewis acids MgCl_2_, ZnCl_2_ and LiCl was investigated. Adduct geometries were optimized and the Gibbs Free Energy of the reaction (Δ*G*_R_) as shown in Scheme 2 was compared. Furthermore, the metal–C bond distances were investigated to obtain additional insight into the strength of the interaction. It should be noted that for the considered reaction, the NHO substitutes a tetrahydrofurane (THF) ligand; the generated complex is neutral, yet a charge separation takes place, locating the positive charge on the NHO ligand. A tetrahedral complex geometry was employed and it was assumed that the metal–Cl bond will resist substitution. THF is a typical solvent for lactone polymerization and the existence of such tetrahedral structures has been demonstrated for closely related NHC complexes such as ZnCl_2_(SIMes)(THF) [4]. The results for MgCl_2_, ZnCl_2_, and LiCl are shown in Table 1, Table 2 and Table 3, respectively.

From these data, a number of conclusions can be drawn. As a consequence of the strong polarization of the olefinic double bond, the NHOs coordinate preferably with the exocyclic carbon atom to Mg^2+^ or Zn^2+^ (η^1^ bonding). This is in accordance with experimental findings; end-on rather than side-on coordination is typical for NHOs and has been described for a range of different metal complexes [13,17,18,33]. Hence, the parameters *d*_1_ and *d*_2_, defined as the distances of the metal to the exo- and endocyclic olefinic carbon (Scheme 2), can serve as a direct probe for any degree of side-on coordination. Interestingly, a direct comparison immediately reveals differences in this respect for the magnesium-, zinc- and lithium-complexes. While the MgCl_2_ adduct (Table 1) displays a clear preference for end-on binding, this property is even more pronounced for the ZnCl_2_-based analogue (Table 2), where the corresponding Δ*d* is largest although the average bonding distances *d*_1_ and *d*_2_ are even somewhat smaller than found for the series of MgCl_2_-complexes. In contrast, the LiCl series displays larger *d* values, implying a rather weak coordination. Also, the asymmetry between *d*_1_ and *d*_2_ is milder and in some cases the lithium takes up a virtually equidistant position relative to the two olefinic carbons. This effect is obviously influenced by the chemical structure of the NHO and most pronounced for **4**, which may be explained by the relatively low double bond-polarization in this molecule (see below). The increasing tendency to form end-on coordinated adducts in the sequence Li^+^ < Mg^2+^ < Zn^2+^ as described by *d*_1_/*d*_2_ is coherently mirrored by the Gibbs Free Energy of the reaction, which shows the adduct formation to be increasingly exothermic in the same sequence. 

This is true for the full range of all eight investigated NHOs, suggesting that for any given system the proportion of free NHO is highest for LiCl and lowest for ZnCl_2_ (for the latter, the reaction enthalpy is by 15 kJ/mol smaller on average than found for MgCl_2_). The stronger tendency of ZnCl_2_ than MgCl_2_ to bind to the investigated NHOs is also expressed in shorter Zn-C bond lengths (compare Table 1 and Table 2, on average 0.15 Å shorter), in spite of the very similar ionic radii of Mg^2+^ and Zn^2+^. The binding to Li^+^ is unfavorable compared to the binding affinity to Mg^2+^ by up to 45 kJ/mol and compared to ZnCl_2_ by up to 65 kJ/mol, depending on the NHO. In this context it should be noted that the same tendency seems to apply for analogous NHC complexes (MgCl_2_/ZnCl_2_) [4], supporting the findings in this work.

The chemical structure of the NHO significantly influences the reaction energies, allowing for some instructive comparisons. The results are more clearly cut for the zinc- and magnesium analogues—not surprisingly, since here a proper bond is formed—while for the LiCl complexes the weak interaction equalizes the reaction energies. Thus, for compounds **1**–**3**, a series of NHOs with no further substituent on the exocyclic carbon (C=CH_2_ moiety), the bonding propensity increases on going from **1** over **2** to **3**. This nicely matches literature reports, which indicate that a six-membered, tetrahydropyrimidine-based structure such as **2** should be somewhat more reactive than **1** [34], while imidazole derivatives (**3**) display a striking reactivity on account of their extreme double bond polarization (in C_6_D_6_, **3** shows a shift of δ = 2.77 ppm for its olefinic protons [21]). This behavior can be explained by the formation of an aromatic imidazolium moiety once charge separation occurs for NHOs such as **3** or **6** (see also Figure 1b). Reports consequently describe a very different behavior in organopolymerization, where imidazole-based NHOs frequently are able to polymerize while the saturated analogues remain inactive, as was found for lactones, epoxides or acrylic monomers [14,31,35]. The increased electron density on the exocyclic carbon should therefore render NHO **3** most prone for coordination to a Lewis acid, a conclusion that is supported by the data listed in Table 1 and Table 2; the ZnCl_2_(**3**)(THF) adduct accounts for the most exothermic reaction energy for all investigated NHO/metal halide combinations (−53.4 kJ/mol). The series **4**–**6**, analogous to **1**–**3** but with additional dimethyl-substitution on the exocyclic carbon (C=C(CH_3_)_2_ moiety) is of significance since this modification is a prerequisite for successful polymerization in lactone or even acrylate polymerization [31,35]. This also shows in the binding propensity, which is in all three cases much less pronounced than for the non-substituted compounds. For **4** and **5** (MgCl_2_, ZnCl_2_) the reaction energies even turn endothermic, while for the imidazole-derivative **6** still a negative free reaction energy was calculated. Obviously, the increased steric congestion disfavors adduct formation (see Table 4 for buried volumes, %*V*_Bur_, of NHOs **1**–**8**) [36,37]. Since compounds **4**–**6** have been highly successful in the Lewis pair polymerization of lactones [11,12], including the polymerization of VL, CL and PDL as well as the copolymerization of GBL, this destabilization of adducts seems to be beneficial for catalytic activity. Moving to compound **7**, a benzimidazole derivative, the data from MgCl_2_ and ZnCl_2_ coordination put this NHO in an intermediate range between the less polarized saturated ring systems and the imidazole structure **6**. This suggests that benzimidazole backbones might be suitable to realize polymerization systems where the NHO is still considerably active, but does not form too strongly associated Lewis pairs. Experimental data for this NHO is scarce, but a report on VL/CL homo- and copolymerization (in cooperation with Lewis acids such as MgCl_2_, ZnCl_2_, YCl_3_) attests to a high catalytic performance with good control over polydispersity (*Ð*_M_ = 1.1) [11]. Finally, NHO **8** shall be considered. For this saturated, five-membered compound a seemingly small change (connecting the exocyclic methyl substituents to form a cyclopropane) can be observed to have a surprising impact. Comparing the reaction energies for **1**, **4**, and **8** (ZnCl_2_) of −26.6 kJ/mol, +7.6 kJ/mol and −44.1 kJ/mol, respectively, reveals that the bonding propensity for this particular NHO is the strongest in this series and even more pronounced than for imidazole derivative **6** (for MgCl_2_ the same applies). This is striking; potentially, the electron donating effect exerted by the cyclopropane moiety overcompensates for steric crowding (according to Table 4 the steric demand of **8** is intermediate between NHOs **1** and **4**) and for having a saturated backbone in the heterocyclic ring. ^13^C-NMR investigations of this compound attest to the high electron density on the exocyclic, olefinic carbon [20], which can be interpreted to support the calculated propensity for complex formation. Information on the polymerization activity of this NHO does currently not exist, but a high activity in organopolymerization might be expected on the basis of these findings. 

Overall, the results described in this section indicate that adduct formation for NHO/metal halides Lewis pairs can be readily influenced—perhaps even tailored—by suitable selection of both components. For example, the **4**/LiCl LP can be expected to offer a high degree of “free” cocatalysts, while the application of **3**/ZnCl_2_ should provide a result from the other end of the scale. Furthermore, it was found that the exocyclic substituents are a prime tuning site for regulating adduct formation. The good congruency of experimental findings with the tendencies described in this section also suggest that future NHO (co)catalysts could be conveniently screened in this manner, with a limited computational effort (see Computational Details).

### 2.2. Coordination of Lactones to MgCl_2_

Another central assumption in the discussed type of LP lactone polymerization is the activation of the monomer by the Lewis acid. As a representative example we have chosen MgCl_2_ and studied its interaction with β-butyrolactone (BBL), GBL, VL and CL in different coordination geometries and activation modes (Figure 2). The coordination geometries include (distorted) tetrahedral and trigonal-bipyramidal configurations, while the activation modes include lactone-metal interactions via the endocyclic oxygen, via the carbonyl oxygen and a chelating binding motif employing both oxygens. By choice of the authors, octahedral complexes were not considered; the similar, although not identical, situation with NHC-MgCl_2_ complexes suggested low coordination numbers [4]. For all models it was assumed that the Mg-Cl bonds remain intact and free coordination space is taken up by THF (one solvent molecule for the tetrahedral coordination, two THF molecules for trigonal-bipyramidal configuration). Interaction of the lactone with MgCl_2_ in the pentacoordinate scenario always resulted in the apical positioning of the monomer being preferred.

For the optimized structures, results are given in Table 5 and Table 6. The distances of Mg to the relevant oxygen atoms are listed, whereby *d*_3_ and *d*_4_ describe the distance to the endocyclic and carbonyl oxygen, respectively. For VL and CL, no minimum structure for the endocyclic binding motif could be found in case of the tetrahedral [MgCl_2_(Lactone)(THF)] complex. All attempts to identify this local minimum resulted in the bidentate structure. This implies, before any quantitative evaluation is considered, that the endocyclic type of activation is energetically disfavored. Likewise, no minimum structure for this coordination was obtained for VL and GBL in the trigonal-bipyramidal configuration, i.e., [MgCl_2_(Lactone)(THF)_2_].

Noticeably, the distances between magnesium and the oxygen atoms are slightly longer for the four-membered BBL, compared to other lactones. This suggests weaker binding to Mg^2+^, an assumption that is also mirrored by the corresponding Δ*G*_R_ values (see below). Generally, for all lactones the O–Mg distances are larger in the trigonal-bipyramidal coordination, which most probably reflects the increased steric hindrance caused by the fifth ligand. Interestingly, also a preference for certain binding motifs becomes apparent. Coordination via the endocyclic oxygen alone is clearly disfavored. Even where such species were identified, *d*_3_ is relatively long with 2.16–2.17 Å (tetrahedral) and 2.39–2.64 Å (trigonal-bipyramidal). For comparison, the bond length to THF in these complexes is 2.08 Å and 2.10/2.20 (equatorial/apical), respectively. This implies a rather weak bonding, which is potentially caused by the reduced electron density on this oxygen atom compared to its carbonyl counterpart and the steric demand exerted by the neighboring carbonyl group, impairing coordination (see bottom left structure, Figure 2). For the bidentate activation mode, only rather high *d*_3_ values were found (2.79–3.45 Å, 3.10–3.47 Å), while the distance to the carbonyl oxygen (*d*_4_) was in the range of 2.09–2.10 Å and 2.17–2.22 Å, respectively, for the two geometries investigated. While this negates a truly chelating coordination of the lactone, it is nonetheless interesting to note that the Mg–O (*d*_3_) interaction significantly grows upon increasing the ring size of the monomer, as evidenced by the shrinking interatomic distances reported in Table 5 and Table 6. 

The most probable explanation for this behavior is found in the required four-membered cycle (Mg–O–C–O), which is clearly strained. However, this strain lessens when the O–C–O angle gets smaller; consequently, for BBL with its rigid structure and largest O-C-O angle an effective bidentate activation is clearly disfavored, while CL with its smaller corresponding angle and higher flexibility can achieve a distance *d*_3_ of 2.79 Å, indicating a weak interaction. The third mode of activation, exclusively via the carbonyl oxygen, delivers the shortest Mg-O distances of all investigated binding motifs, suggesting it as the preferred coordination type for the lactone. This is also supported by another indicator for the strength of the binding of the lactones to magnesium, namely the binding energy derived from the Gibbs Free Energy of the reaction [39,40,41]:

[MgCl_2_(THF)_x_] + Lactone → [MgCl_2_(THF)_x−1_(Lactone)] + THF

Geometries as depicted in Figure 2 were employed. The results of these calculations are summarized in Table 7.

The Gibbs Free Energy of the reaction for BBL is generally higher than the corresponding data of the other lactones, implying that BBL is less likely bound and thereby also less likely to be activated for ring-opening in this manner. Albeit circumstantial, it is still noteworthy that so far no reports for BBL polymerization using this type of LP exist, perhaps a consequence of the seemingly ineffectual activation. 

For all lactones, the endocyclic binding mode displays a rather large average reaction energy of +18.3 kJ/mol. Together with the findings on Mg–O bond lengths (see above) this suggests that this mode of coordination can be considered less likely. For the bidentate binding mode, the average reaction energy (+5.3 kJ/mol) is slightly higher than the average reaction energy of the carbonyl-only motif (+3.6 kJ/mol), thus the latter type of activation is again rendered the most favored one. Hence, overall an endocylic-only coordination can be discounted, while activation via the carbonyl is most likely. Tentatively, this can be correlated with experimental observations. A YCl_3_(CL)_3_ complex has been characterized by crystal data analysis [42], whereby all three CL ligands in this complex are exclusively coordinated via the carbonyl oxygen. However, it is unclear whether the same applies in solution. A chelate-like coordination by the lactone cannot be ruled out by the results discussed above. The stepwise increasing ability of the lactone to achieve coordination via both oxygen species as the ring size grows from the four-membered BBL to the seven-membered CL (especially tetrahedral coordination, see Table 5) could indicate that this is one of the mechanisms by which monomer selectivity is engendered in the NHO/metal halide LP polymerization setup.

### 2.3. Initiation: Proton Transfer Step

**Scheme 3:**
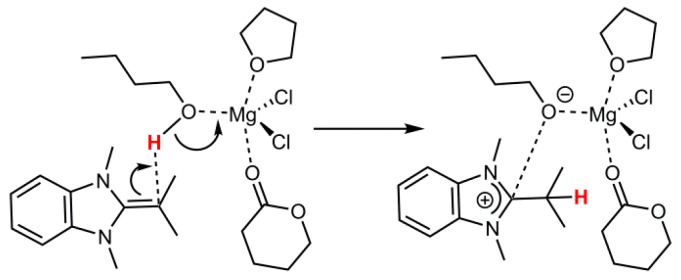
Model employed for calculation of the proton transfer.

**Figure 3:**
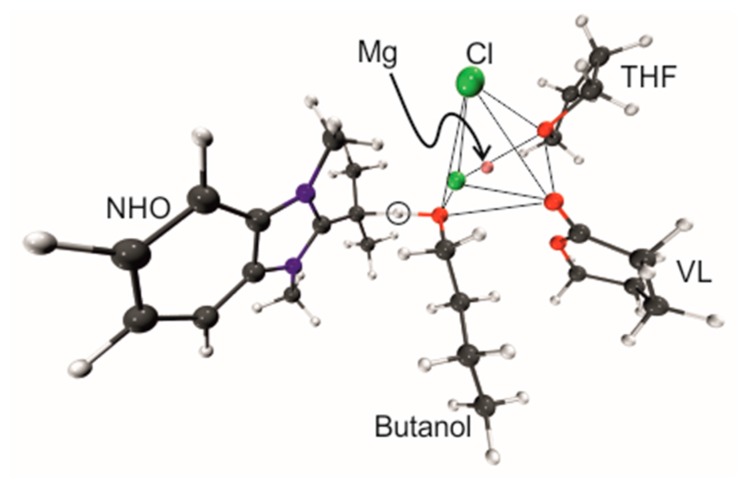
Transition structure of the proton transfer. The lines indicate the trigonal-bipyramidal coordination polyhedron. Encircled: Transferred proton.

Finally, the initial step of the polymerization process, which is often described as the deprotonation/activation of an initiating alcohol species, was also investigated. As model components, the interaction of MgCl_2_ with one VL and one THF molecule in the presence of butanol was investigated. As NHO compound **7** was selected (Scheme 3, Figure 3), since this structure represents intermediate polarization. Also, the successful polymerization of VL by **7**/MgCl_2_ has been reported in the presence of an alcohol as initiator (conversion > 90%, 80 min, 1% NHO-loading, *Ð*_M_ = 1.10–1.20) [11].

Calculations revealed a potential activation energy barrier of 30.2 kJ/mol for the proton transfer (20.9 kJ/mol including zero point energy (ZPE)) and a potential reaction energy of −14.5 kJ/mol (−13.3 kJ/mol including ZPE), rendering this process well feasible. However, *without* MgCl_2_ being present, the deprotonation of butanol with NHO **7** was calculated to be endothermic by approximately 65 kJ/mol (see also Figure S1, Supplementary Materials). Significantly, this is in accordance with recent experimental descriptions of the deprotonation of benzyl alcohol, a typical initiator for lactone polymerization, where it was detailed that in the absence of metal-based Lewis acids no proton transfer was observed (^1^H-NMR), yet with different metal halides varying proportions of protonated NHO species were detected [12]. Thus, especially less reactive NHOs (such as **1**, **2**, **4**, **5** or **7**) seem to require the additional support by a Lewis acid to efficiently polymerize lactones, most probably because the electron deficient metal species stabilizes the formation of an oxyanionic species (butanolate) from the initiator and thus acidifies the –OH functional group.

During the course of the proton transfer the coordination sphere of Mg^2+^ remains broadly unchanged. The Mg-O_butanol_ distance (Table 8) shrinks from 2.13 Å to 1.95 Å, nicely mirroring the stronger bonding of the butanolate to Mg^2+^ (as compared to the initial butanol). The olefinic C=C double bond in the NHO is converted into a single bond upon protonation, which is reflected by the changing C–C distance, growing from 1.39 Å to 1.50 Å. 

## 3. Computational Details

All density functional calculations were carried out using Turbomole v. 7.0.1 [43]. Geometry optimizations have been performed using the dl-find optimization library interfaced via ChemShell with default convergence criteria [44]. For geometry optimizations, the BP-86 functional [45,46,47,48,49] in combination with the def2-SVPD basis set [50] was used throughout. To account for the solvation, which is crucial for the stabilization of the partial charges occuring, i.e., for the adduct formation and the proton transfer reaction, the Conductor-like Screening Model (COSMO) [51] with the dielectric constant of ε(THF) = 7.58 was applied in these cases (Section 2.1 and Section 2.3). To improve the quality of the potential energy, single point calculations using the M06-2X functional [52] in combination with the def2-TZVPD basis set have been performed on all structures, as this functional was shown to perform well [53,54]. Test calculations showed that for the adduct formation of NHO **1** to MgCl_2_ (see Scheme 2) the potential reaction energy of 13.2 kJ/mol obtained at M06-2X/def2-TZVPD//M06-2X/def2-SVPD level deviates just slightly from the potential reaction energy of 14.2 kJ/mol obtained by M06-2X/def2-TZVPD//MP86/def2-SVPD. All self-consistent field (SCF) cycles were iterated until the electronic energies changed by less than 10^-9^ Hartree between two successive iterations. The m5 multi-grid was used [55]. Zero point energies and thermal corrections were calculated in the approximation of harmonic oscillators at 300 K. In Section 2.1 and Section 2.2, Gibbs Free Energies are given and in Section 2.3 sole potential energies (on M06-2X/def2-TZVPD level) are provided. 

## 4. Conclusions

Adduct formation, a major factor in Lewis pair polymerization, has been shown to be readily tunable in the case of NHO/metal halide co-catalysts. Among the three investigated Lewis acids, the bonding propensity is strongest for ZnCl_2_, followed by MgCl_2_ and LiCl, with the latter displaying only a weak interaction. In the same order the formation of true end-on coordination recedes. The ability to efficiently form adducts can also be influenced on the NHO side. In general, saturated NHO backbones reduce the polarization of the olefinic bond and likewise disfavor adduct formation. Similarly, the introduction of substituents on the exocyclic olefinic carbon can destabilize NHO/metal halide complexes on account of the increased steric congestion.

Lactone activation via coordination to a Lewis acid is a key motif for many proposed LP polymerization mechanisms. According to the findings presented in this work, if this coordination occurs it will most likely happen via the carbonyl oxygen. An endocyclic-only coordination can be ruled out. Interaction via both oxygen species in the lactone seems to gain importance for the larger-ring monomers, potentially suggesting one possible mode by which monomer selectivity is generated. 

The presence of a metal species also influences the acid-base behavior of NHO and alcohol-based initiators. NHOs of only intermediate polarization cannot deprotonate –OH functionalities in a metal-free setup. As detailed for **7**/MgCl_2_, this changes in the presence of the Lewis acid, where then a well feasible, exothermic process with a low barrier could be identified. These observations are in accordance with the (limited) experimental data available so far, suggesting that the computational evaluation of NHO/metal halide pairs can usefully complement experimental screenings.

## Figures and Tables

**Table 1:** Properties of the MgCl_2_(NHO)(THF) complexes.

NHO	Δ*G*_R_ (kJ/mol)	*d*1 (Å)	*d*2 (Å)
**1**	−9.0	2.26	2.96
**2**	−17.1	2.25	3.04
**3**	−32.3	2.24	3.02
**4**	+20.0	2.31	2.90
**5**	+20.9	2.36	2.93
**6**	−9.6	2.27	2.99
**7**	+12.4	2.34	2.90
**8**	−21.8	2.21	3.09

**Table 2:** Properties of the ZnCl_2_(NHO)(THF) complexes.

NHO	Δ*G*_R_ (kJ/mol)	*d*1 (Å)	*d*2 (Å)
**1**	−26.6	2.09	2.88
**2**	−30.7	2.08	2.89
**3**	−53.4	2.07	2.95
**4**	+7.6	2.13	2.85
**5**	+11.0	2.16	2.84
**6**	−25.3	2.10	2.85
**7**	+2.3	2.15	2.82
**8**	−44.1	2.06	2.92

**Table 3:** Properties of the LiCl(NHO)(THF)_2_ complexes.

NHO	Δ*G*_R_ (kJ/mol)	*d*1 (Å)	*d*2 (Å)
**1**	+15.2	2.38	2.81
**2**	+11.9	2.36	2.86
**3**	+12.9	2.33	3.00
**4**	+29.9	3.00	2.94
**5**	+17.6	2.83	3.12
**6**	+14.2	2.54	2.77
**7**	+27.2	2.68	2.92
**8**	+10.0	2.31	2.79

**Table 4:** Buried volume as calculated for MgCl_2_(NHO)(THF) [38].

NHO	**1**	**2**	**3**	**4**	**5**	**6**	**7**	**8**
%*V*_Bur_	23.4	23.1	22.7	30.5	31.6	29.7	30.2	26.6

**Table 5:** Distances between magnesium and relevant oxygen atoms of the coordinated lactones in the tetrahedral [MgCl_2_(Lac)(THF)] structures. Data in (Å).

Lactone	*d*_3_			*d*_4_		
Binding Mode	Endocycl.	Carbonyl	Bidentate	Endocycl.	Carbonyl	Bidentate
BBL	2.16	4.26	3.45	3.67	2.08	2.10
GBL	2.17	4.21	3.22	3.43	2.07	2.09
VL	-	4.17	2.96	-	2.05	2.09
CL	-	4.18	2.79	-	2.05	210

**Table 6:** Distances between magnesium and relevant oxygen atoms of the coordinated lactones in the trigonal-bipyramidal [MgCl_2_(Lac)(THF)_2_] structures. Data in (Å).

Lactone	*d*_3_			*d*_4_		
Binding Mode	Endocycl.	Carbonyl	Bidentate	Endocycl.	Carbonyl	Bidentate
BBL	2.39	4.43	3.47	3.94	2.24	2.22
GBL	-	4.31	3.30	-	2.17	2.19
VL	-	4.30	3.10	-	2.16	2.17
CL	2.64	4.29	3.10	3.54	2.15	2.18

**Table 7:** Reaction energies (Δ*G*_R_) for the substitution of a THF molecule by a lactone. Data in (kJ/mol).

Lactone	Tetrahedral Complex			Trigonal Bipyramidal Compl.		
Binding Mode	Endocycl.	Carbonyl	Bidentate	Endocycl.	Carbonyl	Bidentate
BBL	+15.1	+6.6	+10.7	+28.0	+25.5	+28.8
GBL	+16.9	−0.4	+1.5	-	−8.3	+4.6
VL	-	−6.6	−7.8	-	−0.6	+2.9
CL	-	+12.5	+7.1	+13.1	−0.1	−5.1

**Table 8:** Relevant interatomic distances changing during the proton transfer step. All values in (Å).

Parameter	Alcohol Coordinated	Deprotonated
*d*(O_butanol_–H)	1.01	1.92
*d*(C_NHO_–H)	1.98	1.13
*d*(Mg–O_butanol_)	2.13	1.95
*d*(C=C)	1.39	1.50

## Supplementary Materials

The following are available online. molecular coordinates for various structures, TAR-archive for DFT structures, Figure S1: Proton transfer in the absence of MgCl_2_.
