# Supplementary material for: The Lewis Pair Polymerization of Lactones Using Metal Halides and N-Heterocyclic Olefins: Theoretical Insights
Source: Molecules. 2018 Feb 15;23(2):432. doi: 10.3390/molecules23020432 (PMC6017504; doi:10.3390/molecules23020432)
Supplement: Supplementary file 1 [file molecules-23-00432-s001.zip › Molecules_SI_2.0.pdf]

Supporting Information to

The Lewis Pair Polymerization of Lactones Using Metal Halides and N-Heterocyclic Olefins: Theoretical Insights

Jan Meisner [1], Johannes Karwounopoulos[1], Patrick Walther [2], Johannes Kästner[1] and Stefan Naumann[2,*]

[1] Institute of Theoretical Chemistry, University of Stuttgart, Pfaffenwaldring 55, D-70569 Stuttgart, Germany;
[2] Institute of Polymer Chemistry, University of Stuttgart, Pfaffenwaldring 55, D-70569 Stuttgart, Germany;
* Correspondence: stefan.naumann@ipoc.uni-stuttgart.de, Tel.: +49 0711 685 64090.

Molecular structures 2
THF 2
NHOs 2
NHO Adducts 8
Lactones 36
Tetrahedral [$MgCl_2$(THF)(Lac)] complexes 38
Trigonal-bipyramidal [$MgCl_2$$(THF)_2$(Lac)] complexes 45
Structures related to the proton transfer reaction 56
Details to the calculations of the metal-free deprotonation 61
References 62

Molecular structures

Molecular structures of all calculated stationary points and minima (transition structures) were ensured to have no (one) imaginary vibrational frequency larger than 50i cm^{-1}.

THF

13

H -1.571636 1.288654 -1.100552
H -4.072959 1.209388 -2.335639
H -3.058522 0.979955 -0.134227
C -2.437940 0.609211 -0.979598
H -2.627407 0.537420 -3.154682
C -3.271775 0.451616 -2.257315
O -1.942723 -0.711339 -0.656422
C -3.807510 -0.984890 -2.114469
H -4.689104 -1.004720 -1.443492
H -4.099189 -1.439141 -3.079280
C -2.620094 -1.703729 -1.462339
H -1.913065 -2.093614 -2.227522
H -2.920416 -2.543173 -0.805194

NHOs

NHO 1:
20

C -3.803223 1.761383 0.289783
C -4.558486 0.445539 0.494028
N -2.464506 1.270280 -0.042913
N -3.893805 -0.418458 -0.484202
C -2.574100 0.018365 -0.661074
C -1.577263 -0.653472 -1.317067
H -0.592881 -0.196502 -1.462536
H -1.744176 -1.660890 -1.712271
C -1.430442 2.207382 -0.427723
C -4.226529 -1.826863 -0.534174
H -1.493887 3.099093 0.221385
H -1.520904 2.532690 -1.490461
H -0.430348 1.756302 -0.285796
H -5.320673 -1.942064 -0.433817

H -3.730563 -2.412017 0.275281
H -3.929090 -2.258432 -1.508199
H -4.247955 2.349283 -0.548736
H -3.781057 2.400205 1.191976
H -4.424323 0.068032 1.536180
H -5.641693 0.523371 0.286707

NHO 2:
23
Energy = 0.00 kcal/mol
C -3.656110 2.286985 0.374247
C -5.058593 0.238957 0.358321
N -2.495047 1.433630 0.099002
N -3.841518 -0.532205 0.082102
C -2.713343 0.143665 -0.422453
C -1.832277 -0.451861 -1.295982
C -1.330019 2.159400 -0.380278
C -4.094749 -1.874088 -0.416830
H -1.258510 3.112941 0.171470
H -1.379882 2.390063 -1.471127
H -0.406604 1.581233 -0.191653
H -4.960137 -2.293220 0.125610
H -3.222621 -2.528505 -0.233415
H -4.323206 -1.896035 -1.509103
H -0.926179 0.059815 -1.629395
H -1.978158 -1.477521 -1.643344
C -4.728320 1.511100 1.127835
H -4.082676 2.698299 -0.574835
H -3.306131 3.150968 0.970040
H -5.637125 2.132522 1.238663
H -4.362713 1.252979 2.141525
H -5.741558 -0.406130 0.942650
H -5.590104 0.498332 -0.591411

NHO 3:

24
Energy = 0.00 kcal/mol
C -3.813551 1.690036 0.383138
C -4.576888 0.548081 0.376609
C -5.953065 0.294866 0.894960
H -6.365986 1.215525 1.342034
H -5.969476 -0.492858 1.677697
H -6.650547 -0.028268 0.093343
C -4.106464 3.053590 0.913712
H -4.066160 3.830518 0.121196
H -3.389543 3.358695 1.705253
H -5.118071 3.081115 1.353976

N -2.584242 1.374490 -0.236056
N -3.812780 -0.461829 -0.248404
C -2.574860 0.041745 -0.635517
C -1.556917 -0.633781 -1.279724
C -1.470889 2.258697 -0.486801
C -4.189765 -1.835985 -0.480652
H -1.683945 3.259203 -0.078361
H -1.279030 2.353164 -1.576622
H -0.546285 1.872009 -0.009559
H -5.205526 -2.018857 -0.095795
H -3.487571 -2.528558 0.029332
H -4.174518 -2.071767 -1.565522
H -0.621755 -0.124062 -1.531936
H -1.666785 -1.690210 -1.543894

NHO 4:
26
Energy = 0.00 kcal/mol
C -3.635339 1.668150 0.520071
C -4.555036 0.461336 0.445459
N -2.661872 1.405106 -0.552690
N -3.655098 -0.608317 -0.015722
C -2.590454 -0.004469 -0.743645
C -1.653187 -0.668630 -1.497020
C -0.785390 0.028307 -2.523368
H -1.236396 0.965671 -2.898347
H -0.657305 -0.637551 -3.402376
H 0.242642 0.263142 -2.163918
C -1.406867 -2.156451 -1.365508
H -1.898727 -2.769856 -2.154934
H -1.718458 -2.555021 -0.382164
H -0.318332 -2.353412 -1.459320
C -1.424746 2.162046 -0.367629
C -4.389334 -1.721291 -0.615036
H -1.687146 3.211575 -0.141425
H -0.798052 2.162948 -1.271819
H -0.821672 1.757801 0.478910
H -5.209193 -2.008576 0.068538
H -3.753658 -2.606540 -0.764088
H -4.832831 -1.436150 -1.597905
H -5.025680 0.205813 1.413405
H -5.368100 0.632151 -0.299943
H -4.158388 2.630545 0.365509
H -3.120301 1.709353 1.509429

NHO 5:

29
Energy = 0.00 kcal/mol
C -3.920622 2.264719 -0.029433
C -4.675481 0.086537 0.913881
N -2.695726 1.628864 -0.531147
N -4.006194 -0.335892 -0.318676
C -2.803514 0.229070 -0.760137
C -1.832421 -0.476862 -1.442805
C -1.476579 2.104675 0.124332
C -4.655499 -1.400057 -1.060927
H -1.423425 1.823231 1.202683
H -1.433341 3.207406 0.050320
H -0.583027 1.694876 -0.370952
H -5.745723 -1.196592 -1.119845
H -4.532487 -2.408526 -0.606906
H -4.243501 -1.430971 -2.084249
C -0.823723 0.210232 -2.331929
C -1.685286 -1.977606 -1.338042
C -4.384540 1.563503 1.253451
H -4.710431 2.207294 -0.805919
H -3.711713 3.336516 0.145599
H -5.286253 2.044288 1.679597
H -3.590006 1.647332 2.020683
H -4.409572 -0.576769 1.770394
H -5.763715 -0.049567 0.746747
H -0.621108 -2.241914 -1.153493
H -1.972408 -2.519237 -2.268737
H -2.272435 -2.403843 -0.505305
H 0.195805 0.276316 -1.883389
H -1.139146 1.237082 -2.598190
H -0.695632 -0.361578 -3.276744

NHO 6:
30
Energy = 0.00 kcal/mol
C -3.993544 1.654836 0.250773
C -4.627478 0.460011 0.451467
C -5.905111 0.158759 1.163231
H -6.285355 1.064099 1.667394
H -5.766497 -0.624664 1.936919
H -6.706040 -0.197899 0.481362
C -4.364336 3.027007 0.708544
H -4.400095 3.743630 -0.138433
H -3.652904 3.441962 1.453425
H -5.361873 3.016831 1.181165
N -2.824442 1.407112 -0.514147
N -3.856080 -0.554811 -0.170229

C -2.725467 0.025081 -0.782529
C -1.729217 -0.619135 -1.496299
C -0.857737 0.095723 -2.506774
H -1.255647 1.083206 -2.801330
H -0.793925 -0.510005 -3.438508
H 0.195894 0.243643 -2.172973
C -1.419465 -2.091523 -1.333293
H -1.694912 -2.709306 -2.219665
H -1.901350 -2.543510 -0.448274
H -0.322821 -2.231598 -1.200702
C -1.647736 2.261414 -0.442378
C -4.432921 -1.807068 -0.634373
H -1.792307 3.028334 0.337301
H -1.429545 2.778649 -1.396756
H -0.766533 1.646899 -0.171727
H -5.532109 -1.764577 -0.550448
H -4.078206 -2.686146 -0.061186
H -4.168703 -1.957311 -1.699448

NHO 7:
30
Energy = 0.00 kcal/mol
C -3.861766 1.892710 0.206380
C -4.647426 0.715407 0.323376
C -5.897058 0.739849 0.953767
C -4.307542 3.115791 0.721385
N -2.683965 1.548470 -0.454365
N -3.943539 -0.321704 -0.286441
C -2.709463 0.168548 -0.789742
C -1.741732 -0.535666 -1.468240
C -1.464253 2.328910 -0.301830
C -4.608668 -1.522160 -0.770264
H -1.564370 2.979387 0.586461
H -1.249750 2.970885 -1.177835
H -0.611518 1.646490 -0.138826
H -5.697374 -1.337020 -0.808608
H -4.431448 -2.402837 -0.123184
H -4.259130 -1.750310 -1.792937
C -0.697734 0.132601 -2.337990
C -1.620375 -2.043976 -1.420882
C -5.570800 3.140084 1.362243
C -6.351845 1.978092 1.468848
H -7.331135 2.024662 1.966538
H -6.503993 -0.169695 1.061751
H -3.710684 4.032244 0.621081
H -5.945360 4.088045 1.773531
H -1.896417 -2.535726 -2.380835

H -2.210763 -2.512580 -0.615511
H -0.558464 -2.317290 -1.236263
H -0.635319 -0.406138 -3.308636
H 0.326888 0.094033 -1.904336
H -0.927943 1.185214 -2.575550

NHO 8:
24
Energy = 0.00 kcal/mol
C -3.035487 2.004495 0.463173
C -4.112326 0.919400 0.449518
N -2.241442 1.645498 -0.714448
N -3.367336 -0.227553 -0.075833
C -2.337683 0.251889 -0.912959
C -1.583702 -0.489817 -1.757121
C -0.972666 2.323337 -0.905109
C -4.123585 -1.406948 -0.453686
H -1.135813 3.416541 -0.876399
H -0.548185 2.061867 -1.889403
H -0.228335 2.056378 -0.118139
H -4.756952 -1.721303 0.396303
H -3.436573 -2.234682 -0.699086
H -4.781458 -1.221965 -1.335542
C -0.464322 -0.555130 -2.713002
C -1.261895 -1.814129 -2.316944
H -1.917237 -2.265223 -3.083862
H -0.744112 -2.563083 -1.690409
H 0.577224 -0.476306 -2.352334
H -0.599278 -0.175529 -3.742221
H -3.443112 3.028881 0.375177
H -2.426580 1.946971 1.397650
H -4.529682 0.702165 1.450440
H -4.953116 1.203656 -0.228420

[$MgCl_2(THF)_2$]
29
Energy = 0.00 kcal/mol
Mg -0.398179 1.817296 -0.998489
O 1.310018 2.220883 0.110600
O -1.969209 1.899294 0.361726
C -2.929658 2.994123 0.395753
C -4.265260 2.370689 -0.051811
H -2.540150 3.780750 -0.273335
H -2.977940 3.370032 1.438838
C -4.074521 0.838810 0.162189

H   -4.463024 2.597932 -1.114517
H   -5.107270 2.774495 0.539065
C   -2.687481 0.715013 0.809148
H   -4.091083 0.303421 -0.804294
H   -4.858798 0.398714 0.804110
H   -2.100587 -0.160208 0.481145
H   -2.741548 0.746543 1.917777
C   1.962486 3.529772 0.115282
C   3.453730 3.263192 -0.199129
H   1.821611 3.977702 1.119017
H   1.435297 4.141373 -0.638369
C   3.554397 1.722910 -0.346834
C   2.323473 1.218345 0.403162
H   2.485138 1.191710 1.502096
H   1.924197 0.249772 0.054465
Cl   -0.691582 3.732083 -2.255327
Cl   -0.212619 -0.436737 -1.462409
H   4.093687 3.624938 0.626850
H   3.772856 3.782944 -1.119823
H   3.489425 1.422516 -1.409427
H   4.493755 1.314682 0.067452

NHO Adducts

[MgCl2(THF)(NHO1)]:
36
Energy = 0.00 kcal/mol
C   -3.875946 1.898906 0.268227
C   -4.633682 0.574995 0.439840
N   -2.530840 1.430460 -0.090646
N   -3.853271 -0.329887 -0.415529
C   -2.586498 0.150502 -0.576242
C   -1.482797 -0.582152 -1.086043
H   -0.699666 0.017997 -1.575093
Mg   -0.438467 -1.413363 0.733332
O   0.667806 0.154672 1.509954
H   -1.743942 -1.489598 -1.650530
C   -1.473285 2.371976 -0.398381
C   -4.225017 -1.726327 -0.546970
H   -1.508891 3.201236 0.329987
H   -1.571642 2.797940 -1.419990
H   -0.489372 1.879948 -0.310906
H   -5.323131 -1.792881 -0.641936
H   -3.899675 -2.318536 0.333782
H   -3.774843 -2.155921 -1.459141
C   2.071114 0.398940 1.151991

C 0.310446 0.913905 2.714584
C 2.486190 1.601186 1.996550
H 2.115470 0.569481 0.061561
H 2.650049 -0.511845 1.397576
C 1.640118 1.418167 3.270779
H -0.247114 0.235979 3.383609
H -0.353295 1.745424 2.405268
H 2.094914 0.657762 3.933917
H 1.517782 2.352055 3.847487
H 2.221832 2.547847 1.485763
H 3.572421 1.607134 2.194280
Cl 1.194598 -2.991974 0.118589
Cl -1.952586 -1.937082 2.468346
H -4.304818 2.516499 -0.551471
H -3.850763 2.508620 1.189240
H -4.609714 0.220403 1.492829
H -5.685182 0.624715 0.105823

[MgCl2(THF)(NHO2)]:
39
Energy = 0.00 kcal/mol
C -3.554767 2.370219 0.318735
C -4.850571 0.278997 0.640720
N -2.433171 1.565684 -0.172146
N -3.803610 -0.342807 -0.186543
C -2.598985 0.236333 -0.457982
C -1.512309 -0.533324 -0.995525
Mg -0.298736 -1.399194 0.684126
O 0.719867 0.135955 1.641101
C -1.205102 2.251751 -0.555188
C -4.061259 -1.728106 -0.571668
H -1.227138 3.269365 -0.133496
H -1.097927 2.334029 -1.656460
H -0.316586 1.727836 -0.156003
H -5.136594 -1.929834 -0.436758
H -3.485709 -2.437185 0.057930
H -3.813275 -1.897676 -1.635319
C 2.154236 0.402554 1.480752
C 0.193155 0.865514 2.801332
C 2.417752 1.633115 2.343743
H 2.351812 0.545465 0.404176
H 2.712186 -0.485673 1.833881
C 1.422061 1.442814 3.503502
H -0.390674 0.151845 3.408489
H -0.480893 1.658446 2.421048
H 1.820128 0.721068 4.242085
H 1.188962 2.384095 4.031876
H 2.192787 2.560106 1.780900

H 3.468479 1.680566 2.680119
Cl 1.439083 -2.731744 -0.188977
Cl -1.677784 -2.264704 2.400262
H -0.787177 0.036621 -1.594582
H -1.808251 -1.462212 -1.502314
C -4.361607 1.551313 1.318649
H -4.199628 2.701902 -0.525823
H -3.140995 3.277650 0.792362
H -5.226398 2.130672 1.689465
H -3.721633 1.303081 2.187521
H -5.171223 -0.460985 1.399715
H -5.732607 0.490670 -0.000574

[MgCl2(THF)(NHO3)]:
40
Energy = 0.00 kcal/mol
C -3.624310 1.868022 0.414332
C -4.389155 0.726861 0.511143
C -5.726670 0.504651 1.132616
H -6.094704 1.443162 1.580041
H -5.689927 -0.257440 1.937091
H -6.479188 0.168835 0.390523
C -3.886422 3.257469 0.889614
H -3.910128 3.985888 0.053721
H -3.121549 3.607940 1.612419
H -4.864252 3.300543 1.397957
N -2.451394 1.510704 -0.277958
N -3.670483 -0.300483 -0.125239
C -2.479718 0.179555 -0.596102
C -1.427192 -0.578765 -1.205275
Mg -0.083649 -1.360691 0.413783
O 0.276544 0.262201 1.685147
C -1.347377 2.389218 -0.627863
C -4.077046 -1.691288 -0.267504
H -1.528181 3.389533 -0.206458
H -1.249668 2.477563 -1.726182
H -0.400007 1.993373 -0.217896
H -5.073167 -1.827815 0.180333
H -3.353172 -2.349397 0.250315
H -4.127269 -1.968297 -1.337407
C -0.642719 0.641421 2.764455
C 1.601947 0.850880 1.922087
C 0.243383 1.262014 3.842224
H -1.182755 -0.269188 3.078311
H -1.366598 1.372203 2.351134
C 1.374237 1.895408 3.011630
H 1.968292 1.257808 0.963552

H 2.283941 0.043572 2.250657
H 1.042747 2.856342 2.570685
H 2.290779 2.083919 3.597365
H 0.647243 0.476951 4.510330
H -0.305971 1.993018 4.461140
Cl 2.093529 -1.890978 -0.312896
Cl -1.207782 -2.846456 1.882644
H -0.767929 0.022913 -1.853101
H -1.782565 -1.487252 -1.719078

[MgCl2(THF)(NHO4)]:
42
Energy = 0.00 kcal/mol
C -3.335738 1.833816 0.349984
C -4.058041 0.544857 0.736988
N -2.114254 1.310120 -0.267196
N -3.526790 -0.400227 -0.252628
C -2.290471 0.004939 -0.701892
C -1.311847 -0.759245 -1.416964
C -0.534258 -0.101936 -2.567598
H -0.321421 0.970211 -2.449723
H -1.106768 -0.211018 -3.516783
H 0.433687 -0.616831 -2.724181
Mg 0.059544 -1.322998 0.356480
O 0.907955 0.232358 1.428253
C -1.573366 -2.230081 -1.781335
H -2.234564 -2.338627 -2.669718
H -1.990382 -2.838639 -0.960912
H -0.610443 -2.698264 -2.066318
C -0.992328 2.191803 -0.498372
C -4.208206 -1.671647 -0.426505
H -0.989485 2.962814 0.292403
H -1.036604 2.713668 -1.477221
H -0.041452 1.635286 -0.434994
H -5.288802 -1.494326 -0.278775
H -3.877771 -2.431228 0.311555
H -4.069851 -2.060278 -1.444918
C 2.280392 0.715478 1.237016
C 0.337121 0.800555 2.656478
C 2.390604 1.931542 2.150805
H 2.413353 0.933775 0.163284
H 2.975939 -0.094346 1.527874
C 1.493657 1.532454 3.337621
H -0.091337 -0.031206 3.241982
H -0.475438 1.491710 2.358802
H 2.035511 0.852622 4.022137

H 1.139828 2.400022 3.921921
H 1.995639 2.835093 1.646869
H 3.436033 2.129613 2.445539
Cl 1.971787 -2.362690 -0.536695
Cl -1.244274 -2.392163 2.004557
H -3.802806 0.217280 1.768827
H -5.156340 0.616630 0.650642
H -3.924249 2.439395 -0.374693
H -3.091402 2.472230 1.217770

[MgCl2(THF)(NHO5)]:
45
Energy = 0.00 kcal/mol
C -3.500918 2.238405 0.048713
C -4.396939 0.117429 0.918891
N -2.396681 1.502568 -0.576489
N -3.784826 -0.338076 -0.341930
C -2.585511 0.150262 -0.814566
C -1.611295 -0.657468 -1.488573
Mg -0.223782 -1.138571 0.354316
O 0.634156 0.382450 1.484899
C -1.104320 2.168997 -0.611935
C -4.568735 -1.334569 -1.062275
H -0.980929 2.795573 0.291784
H -0.996224 2.824740 -1.499135
H -0.289326 1.428993 -0.635073
H -5.620361 -0.987773 -1.108976
H -4.562224 -2.327089 -0.567710
H -4.202621 -1.440676 -2.093141
C 1.983931 0.915632 1.249108
C 0.121927 0.856025 2.775245
C 2.191706 1.968074 2.336800
H 2.018395 1.316762 0.220306
H 2.696081 0.073728 1.327334
C 1.339006 1.425454 3.498477
H -0.357757 -0.003655 3.273106
H -0.639149 1.636930 2.575329
H 1.878199 0.623808 4.038392
H 1.056190 2.205008 4.227817
H 1.814727 2.954997 2.005190
H 3.258868 2.077950 2.598735
Cl 1.720411 -2.107409 -0.553709
Cl -1.482397 -2.228580 2.024410
C -0.862970 -0.100325 -2.703017
C -1.810486 -2.173118 -1.621081
C -3.898735 1.506762 1.331653
H -4.363327 2.295279 -0.647669

H   -3.162335 3.269979 0.241889
H   -4.685116 2.047443 1.890547
H   -3.012422 1.436364 1.991414
H   -4.210282 -0.636132 1.712856
H   -5.491060 0.151989 0.748158
H   -0.864202 0.998952 -2.772202
H   -1.342168 -0.474731 -3.635067
H   0.190447 -0.442684 -2.734676
H   -2.395460 -2.452523 -2.524571
H   -2.300095 -2.631452 -0.743676
H   -0.826182 -2.668588 -1.750199

[MgCl2(THF)(NHO6)]:
46
Energy = 0.00 kcal/mol
C   -3.849534 1.677038 0.296806
C   -4.612699 0.538091 0.315728
C   -5.997402 0.324810 0.829532
H   -6.384091 1.265617 1.255933
H   -6.029651 -0.441311 1.629830
H   -6.702096 0.007009 0.034303
C   -4.164733 3.058283 0.764025
H   -4.059284 3.804642 -0.048898
H   -3.511845 3.383084 1.599696
H   -5.205590 3.102044 1.125719
N   -2.618812 1.331878 -0.282550
N   -3.846651 -0.481444 -0.277452
C   -2.598350 -0.008153 -0.630549
C   -1.445734 -0.717786 -1.166562
C   -0.680970 -0.020237 -2.308811
H   -0.410647 1.032730 -2.134562
H   -1.266703 -0.050103 -3.256838
H   0.264341 -0.563052 -2.503965
Mg   -0.144798 -1.140293 0.645420
O   0.847810 0.509442 1.454552
C   -1.613383 -2.197780 -1.564913
H   -2.234338 -2.332689 -2.480069
H   -2.025737 -2.846057 -0.772624
H   -0.615163 -2.606932 -1.811130
C   -1.489006 2.246212 -0.326770
C   -4.362074 -1.841866 -0.416949
H   -1.655438 3.052739 0.403936
H   -1.358985 2.702856 -1.324639
H   -0.566045 1.708268 -0.050028
H   -5.453458 -1.813931 -0.282243
H   -3.929996 -2.508895 0.350674
H   -4.157981 -2.237899 -1.420859

C 2.224901 0.870472 1.075223
C 0.527124 1.100700 2.753886
C 2.796929 1.649262 2.269048
H 2.182457 1.468312 0.145846
H 2.755140 -0.076992 0.871884
C 1.878927 1.247648 3.440821
H -0.185152 0.419595 3.250469
H 0.044784 2.085420 2.582665
H 2.192472 0.279609 3.876457
H 1.857706 2.003208 4.246309
H 2.734668 2.739410 2.089888
H 3.855789 1.392876 2.449389
Cl 1.688279 -2.513553 0.053397
Cl -1.532176 -1.852017 2.424957

[MgCl2(THF)(NHO7)]:
46
Energy = 0.00 kcal/mol
C -3.157430 1.990173 0.140332
C -3.997812 0.876204 0.361592
C -5.155619 0.981959 1.143263
C -3.453126 3.248922 0.679912
N -2.096427 1.547752 -0.648540
N -3.443975 -0.198076 -0.335785
C -2.243209 0.182831 -0.930616
C -1.290324 -0.628237 -1.614508
Mg 0.107741 -1.080149 0.211697
O 0.571489 0.565345 1.377019
C -0.935583 2.379858 -0.918118
C -4.146352 -1.471546 -0.461979
H -0.893572 3.178198 -0.158851
H -0.983066 2.850988 -1.916946
H -0.013171 1.781192 -0.842697
H -5.215367 -1.290752 -0.262813
H -3.767121 -2.214471 0.262942
H -4.056428 -1.860137 -1.485980
C 1.900337 1.192314 1.418853
C -0.230944 1.001880 2.530135
C 1.759372 2.331909 2.422935
H 2.154955 1.512197 0.393394
H 2.630576 0.429309 1.748271
C 0.742928 1.761430 3.429258
H -0.672690 0.099608 2.987190
H -1.042166 1.652858 2.148118
H 1.241095 1.067484 4.132623
H 0.233201 2.543476 4.019128
H 1.357442 3.239071 1.931014

H 2.727349 2.591324 2.886624
Cl 2.175821 -1.745311 -0.661811
Cl -1.086717 -2.411891 1.743596
C -0.529101 -0.054035 -2.816741
C -1.541099 -2.124346 -1.856557
C -4.618212 3.357445 1.465936
C -5.453023 2.245224 1.692953
H -6.354004 2.360239 2.311264
H -5.806607 0.119007 1.332398
H -2.815345 4.122841 0.495729
H -4.878151 4.330158 1.905755
H -2.216361 -2.303933 -2.721863
H -1.933154 -2.674201 -0.985389
H -0.578495 -2.601693 -2.128298
H -1.085274 -0.295908 -3.749915
H 0.468524 -0.524965 -2.908137
H -0.382019 1.034774 -2.814648

[MgCl2(THF)(NHO8)]:
40
Energy = 0.00 kcal/mol
C -3.697241 2.161408 -0.446843
C -4.639312 1.000150 -0.811229
N -2.375181 1.621972 -0.795032
N -3.721556 -0.144336 -0.860371
C -2.427177 0.266041 -0.940315
C -1.297005 -0.600344 -1.072756
Mg -0.666178 -1.565286 0.818047
O -1.341115 -0.476183 2.454673
C -1.185075 2.392494 -0.489092
C -4.203084 -1.489270 -1.113291
H -1.191506 3.339454 -1.060557
H -0.286965 1.816335 -0.755671
H -1.143967 2.637811 0.593426
H -4.465915 -1.641090 -2.180739
H -5.108714 -1.661860 -0.504158
H -3.436347 -2.223157 -0.813351
C -2.774291 -0.414847 2.751810
C -0.562915 -0.376211 3.698809
C -2.879537 -0.609309 4.261917
H -3.273318 -1.204871 2.162149
H -3.144120 0.580653 2.435292
C -1.574654 0.033083 4.768106
H 0.246387 0.354694 3.529870
H -0.117100 -1.368290 3.904407
H -1.673371 1.135081 4.812223
H -1.278737 -0.326130 5.769597

H -2.905769 -1.688028 4.507467
H -3.786441 -0.135189 4.677223
Cl 1.675134 -1.588131 1.058675
Cl -1.820054 -3.614214 1.116462
C -0.198125 -0.344939 -2.087309
C -1.128798 -1.528528 -2.265327
H -1.851566 -1.468669 -3.093512
H -0.736915 -2.542994 -2.096193
H 0.843966 -0.533554 -1.789347
H -0.318019 0.481409 -2.805392
H -3.908678 3.087108 -1.011709
H -3.732162 2.406156 0.637381
H -5.436970 0.835696 -0.064208
H -5.118739 1.147792 -1.802207

[ZnCl2(THF)2]:
29
Energy = 0.00 kcal/mol
Zn -0.691732 -1.327596 1.020063
O 0.381527 0.339457 1.539425
C 0.161896 1.113178 2.767634
C 1.718634 0.603657 0.992752
C 1.545830 1.629432 3.151065
H -0.302361 0.441244 3.509445
H -0.541241 1.934977 2.527649
C 2.229792 1.807389 1.781947
H 1.606661 0.783470 -0.091235
H 2.335666 -0.301339 1.150790
H 1.906411 2.752682 1.304950
H 3.332156 1.813862 1.849067
H 2.083525 0.878345 3.761266
H 1.488721 2.569030 3.728793
Cl 0.704814 -2.915308 0.269401
Cl -2.301999 -1.584539 2.567739
H -1.799257 1.537924 -0.417372
H -4.263758 1.136050 -1.677341
H -3.078528 0.528778 0.362266
C -2.451060 0.654939 -0.541403
H -2.746228 1.222835 -2.625260
C -3.275755 0.666107 -1.828375
O -1.584268 -0.523494 -0.655243
C -3.370239 -0.829655 -2.183889
H -4.152004 -1.325997 -1.577298
H -3.595424 -1.004240 -3.250996
C -1.987812 -1.348922 -1.799342

H -1.241590 -1.198044 -2.603753
H -1.968458 -2.405465 -1.480908

[ZnCl2(THF)(NHO1)]:
36
Energy = 0.00 kcal/mol
C -3.838534 1.913864 0.243498
C -4.605380 0.602648 0.477689
N -2.505617 1.423002 -0.134853
N -3.823381 -0.355067 -0.318578
C -2.573605 0.125262 -0.539369
C -1.470282 -0.635827 -1.053818
H -0.746871 -0.036465 -1.628919
Zn -0.402892 -1.413930 0.562076
O 0.670179 0.222622 1.419554
H -1.782988 -1.529610 -1.615826
C -1.431968 2.338057 -0.470865
C -4.210205 -1.752622 -0.391757
H -1.463868 3.194516 0.224883
H -1.522327 2.725561 -1.507638
H -0.457084 1.834470 -0.355461
H -5.307185 -1.809186 -0.504610
H -3.904842 -2.303928 0.520996
H -3.747622 -2.229657 -1.272864
C 2.110975 0.385831 1.227763
C 0.240767 0.900212 2.645549
C 2.486677 1.564601 2.121187
H 2.286745 0.549332 0.149892
H 2.615943 -0.550941 1.534117
C 1.528778 1.385092 3.314342
H -0.342814 0.178625 3.243519
H -0.417524 1.740793 2.348502
H 1.917788 0.616370 4.009332
H 1.370437 2.316907 3.886056
H 2.293034 2.524309 1.603052
H 3.551549 1.538205 2.413577
Cl 1.291299 -2.844478 -0.035638
Cl -1.687773 -2.079955 2.363842
H -4.278077 2.509822 -0.585415
H -3.783858 2.552481 1.143011
H -4.596117 0.301664 1.547087
H -5.652867 0.640434 0.130075

[ZnCl2(THF)(NHO2)]:

39

Energy = 0.00 kcal/mol
C -3.449147 2.343482 0.447779
C -4.816834 0.281480 0.640629
N -2.395437 1.538144 -0.179999
N -3.783563 -0.356535 -0.191013
C -2.584265 0.218949 -0.464320
C -1.492772 -0.570663 -1.006441
Zn -0.407111 -1.358520 0.586920
O 0.648412 0.240571 1.557323
C -1.166348 2.214825 -0.591089
C -4.071113 -1.732460 -0.594922
H -1.209913 3.257420 -0.238799
H -1.056170 2.228040 -1.693326
H -0.276716 1.728384 -0.149399
H -5.158746 -1.893511 -0.512209
H -3.550051 -2.462177 0.056736
H -3.782804 -1.905959 -1.646600
C 2.097667 0.401441 1.478454
C 0.132679 0.913026 2.752248
C 2.379620 1.652648 2.303544
H 2.365085 0.475922 0.410331
H 2.582387 -0.497632 1.908249
C 1.358994 1.513657 3.449567
H -0.412133 0.166038 3.355841
H -0.579827 1.689376 2.410226
H 1.742891 0.820038 4.221687
H 1.123994 2.474918 3.940127
H 2.181582 2.564314 1.705924
H 3.425043 1.691369 2.658115
Cl 1.333990 -2.737217 -0.011369
Cl -1.710115 -2.112438 2.345541
H -0.785618 0.001836 -1.623984
H -1.822324 -1.469377 -1.546488
C -4.257583 1.473858 1.401355
H -4.106033 2.796589 -0.326662
H -2.962145 3.173108 0.990543
H -5.084295 2.055413 1.846930
H -3.604624 1.123364 2.224217
H -5.196298 -0.480682 1.347377
H -5.668687 0.584631 -0.004604

[ZnCl2(THF)(NHO3)]:
40
Energy = 0.00 kcal/mol
C -3.678582 1.954600 0.378699
C -4.313366 0.786482 0.744524
C -5.567665 0.566955 1.521136
H -6.000529 1.536715 1.819129

H -5.387826 -0.016960 2.446236
H -6.332211 0.025974 0.928001
C -4.042557 3.378735 0.634084
H -4.196962 3.941481 -0.308620
H -3.266031 3.913145 1.217737
H -4.982401 3.428686 1.209063
N -2.542635 1.580771 -0.359604
N -3.549034 -0.269887 0.222202
C -2.472377 0.222169 -0.452948
C -1.419613 -0.542626 -1.082746
Zn 0.111861 -1.018004 0.230649
O 0.409717 0.695709 1.495483
C -1.547028 2.472321 -0.937076
C -3.821414 -1.695653 0.363390
H -1.797096 3.512890 -0.682406
H -1.526287 2.369968 -2.037778
H -0.547033 2.230942 -0.533026
H -4.664699 -1.837344 1.055799
H -2.928676 -2.205459 0.772251
H -4.084577 -2.138693 -0.615459
C 1.710931 1.352470 1.624220
C -0.365985 0.864365 2.725923
C 1.551003 2.300333 2.809838
H 1.930049 1.849198 0.662826
H 2.480933 0.578461 1.808393
C 0.591740 1.516435 3.725065
H -0.730678 -0.132079 3.032909
H -1.230785 1.517082 2.489280
H 1.145071 0.742223 4.290725
H 0.060606 2.158457 4.450205
H 1.092812 3.256022 2.487390
H 2.519403 2.526918 3.290583
Cl 2.200445 -1.253871 -0.706319
Cl -0.419997 -2.615619 1.828564
H -0.941431 0.013232 -1.907018
H -1.781302 -1.518710 -1.448837

[ZnCl2(THF)(NHO4)]:
42
Energy = 0.00 kcal/mol
C -3.479405 1.863028 0.111233
C -4.200534 0.587334 0.534628
N -2.205588 1.334357 -0.389608
N -3.537588 -0.435776 -0.291405
C -2.307390 0.002993 -0.708164
C -1.257248 -0.776239 -1.354927
C -0.516328 -0.086744 -2.521507
H -0.186148 0.945924 -2.343407

H -1.164074 -0.073387 -3.425872
H 0.386489 -0.672869 -2.775567
Zn 0.072559 -1.242863 0.235944
O 0.915818 0.395606 1.311173
C -1.603668 -2.202290 -1.835706
H -2.367617 -2.192273 -2.643644
H -1.934857 -2.891136 -1.042835
H -0.689136 -2.645341 -2.270784
C -1.093381 2.244301 -0.580145
C -4.035443 -1.802797 -0.152955
H -1.189326 3.054303 0.164628
H -1.084067 2.711218 -1.585739
H -0.135724 1.729185 -0.400792
H -5.068443 -1.733715 0.227195
H -3.431601 -2.381597 0.572759
H -4.071221 -2.325622 -1.119897
C 2.344294 0.714531 1.258571
C 0.332874 0.882367 2.565041
C 2.533725 1.798994 2.314991
H 2.577323 1.029853 0.226549
H 2.917611 -0.203745 1.489456
C 1.516385 1.383475 3.394245
H -0.224685 0.045439 3.020462
H -0.371581 1.699372 2.310865
H 1.926150 0.565955 4.017917
H 1.229064 2.215297 4.061878
H 2.279913 2.794353 1.900793
H 3.572479 1.833860 2.689194
Cl 1.978175 -2.250686 -0.569574
Cl -0.972647 -2.355979 1.982799
H -4.051458 0.358677 1.612188
H -5.284366 0.607232 0.326497
H -4.021456 2.402671 -0.695127
H -3.308018 2.566057 0.945758

[ZnCl2(THF)(NHO5)]:
45
Energy = 0.00 kcal/mol
C -4.519315 0.947006 0.126344
C -5.084615 -1.402394 -0.233672
N -3.112882 0.644550 -0.176477
N -3.941084 -1.268320 -1.162740
C -2.844206 -0.490144 -0.890837
C -1.475151 -0.851302 -1.291777
Zn -0.647191 -1.494970 0.592973
O 0.893950 -0.045781 1.129298
C -2.129848 1.438615 0.549439

C -4.145487 -1.961850 -2.433709
H -2.480819 1.595704 1.585420
H -1.987717 2.427322 0.069820
H -1.157300 0.929838 0.593522
H -5.165073 -1.723937 -2.795120
H -4.068365 -3.062592 -2.335920
H -3.433434 -1.610976 -3.191454
C 1.079500 0.319388 2.525710
C 2.228944 -0.234480 0.569721
C 2.221331 -0.580557 2.995425
H 0.116352 0.152229 3.036593
H 1.351763 1.395199 2.570403
C 3.129099 -0.689999 1.741551
H 2.558091 0.733452 0.143003
H 2.141152 -0.983366 -0.234602
H 4.014579 -0.034055 1.826530
H 3.484118 -1.724663 1.592787
H 1.819418 -1.572305 3.273543
H 2.748336 -0.159912 3.870531
Cl 0.688173 -3.375093 0.498673
Cl -1.970531 -1.545734 2.482046
C -0.638517 0.267168 -1.940730
C -1.264507 -2.151783 -2.090234
C -5.127974 -0.280401 0.802441
H -5.059421 1.187633 -0.812571
H -4.546128 1.841805 0.769153
H -6.168475 -0.100431 1.129811
H -4.520608 -0.534828 1.691075
H -5.040268 -2.402715 0.245439
H -6.003376 -1.376873 -0.851879
H -0.616098 1.222127 -1.392234
H -1.021772 0.485074 -2.961589
H 0.410392 -0.063877 -2.060118
H -1.474092 -2.027584 -3.174067
H -1.862123 -2.998214 -1.711679
H -0.206317 -2.458459 -2.011800

[ZnCl2(THF)(NHO6)]:
46
Energy = 0.00 kcal/mol
C -3.783436 1.666606 0.277627
C -4.553818 0.530514 0.300348
C -5.958416 0.343456 0.768815
H -6.362990 1.309520 1.114574
H -6.029328 -0.366855 1.616866
H -6.623733 -0.026495 -0.037022

C -4.103135 3.060210 0.704224
H -3.945444 3.787318 -0.117089
H -3.487393 3.390106 1.565575
H -5.159799 3.126007 1.012875
N -2.543251 1.303012 -0.263282
N -3.773400 -0.503752 -0.242871
C -2.527771 -0.034699 -0.591870
C -1.367278 -0.768712 -1.132625
C -0.599840 -0.027092 -2.252109
H -0.208515 0.967688 -1.992492
H -1.237168 0.092247 -3.156869
H 0.272694 -0.639565 -2.546854
Zn -0.147394 -1.197837 0.523016
O 0.868971 0.497924 1.389733
C -1.636117 -2.193170 -1.670696
H -2.316006 -2.187897 -2.551731
H -2.029222 -2.902582 -0.925338
H -0.672744 -2.612369 -2.012818
C -1.415725 2.226247 -0.311494
C -4.257370 -1.884458 -0.288239
H -1.599727 3.041081 0.405227
H -1.290003 2.667341 -1.316077
H -0.493420 1.701812 -0.012996
H -5.315959 -1.886245 0.007265
H -3.692041 -2.510792 0.423722
H -4.186615 -2.297829 -1.304413
C 2.283264 0.759197 1.092813
C 0.531620 1.010859 2.718655
C 2.781409 1.643463 2.237574
H 2.339016 1.238556 0.098124
H 2.798598 -0.218202 1.049659
C 1.875494 1.220805 3.409554
H -0.129374 0.268033 3.197478
H -0.017474 1.966993 2.590650
H 2.229614 0.270916 3.854748
H 1.818907 1.980499 4.209604
H 2.633529 2.714918 1.999823
H 3.855237 1.482743 2.440827
Cl 1.666616 -2.535811 0.009014
Cl -1.394613 -1.939930 2.339997

[ZnCl2(THF)(NHO7)]:
46
Energy = 0.00 kcal/mol
C -3.181009 1.988276 0.117140
C -4.028900 0.880947 0.330684

C -5.208608 1.000754 1.078763
C -3.483891 3.256562 0.632250
N -2.099248 1.528744 -0.630837
N -3.447014 -0.208112 -0.322859
C -2.239610 0.169259 -0.888470
C -1.241398 -0.659115 -1.539899
Zn 0.136783 -1.008886 0.075000
O 0.578253 0.654218 1.328401
C -0.954208 2.378775 -0.924988
C -4.116967 -1.508313 -0.367468
H -0.959036 3.220524 -0.214740
H -1.002357 2.787500 -1.949677
H -0.017993 1.816672 -0.785137
H -5.167945 -1.353873 -0.076855
H -3.652565 -2.219603 0.337535
H -4.107533 -1.919988 -1.385959
C 1.930167 1.203240 1.437066
C -0.199268 0.981542 2.529105
C 1.800857 2.315203 2.471826
H 2.236074 1.536687 0.430230
H 2.617042 0.399143 1.766792
C 0.771140 1.723544 3.453187
H -0.592267 0.037409 2.945881
H -1.048152 1.618676 2.210836
H 1.264991 1.015033 4.144954
H 0.258050 2.491399 4.059248
H 1.410947 3.239579 2.002055
H 2.768387 2.549715 2.949946
Cl 2.220690 -1.547949 -0.702111
Cl -0.828751 -2.404564 1.650046
C -0.555184 -0.061032 -2.784442
C -1.587715 -2.124685 -1.870489
C -4.665602 3.377654 1.385363
C -5.511524 2.269830 1.605195
H -6.422684 2.395611 2.205948
H -5.868861 0.144520 1.264019
H -2.832056 4.122376 0.461082
H -4.930060 4.355022 1.811657
H -2.314861 -2.202396 -2.707214
H -1.954708 -2.710910 -1.014187
H -0.663278 -2.623869 -2.216953
H -1.258136 -0.084935 -3.645877
H 0.317912 -0.684075 -3.051377
H -0.190121 0.970886 -2.688927

[ZnCl2(THF)(NHO8)]:

40
Energy = 0.00 kcal/mol
C -3.614706 2.046655 -0.419973
C -4.498516 0.782875 -0.429113
N -2.257971 1.487602 -0.489520
N -3.564787 -0.263404 -0.871364
C -2.282679 0.176027 -0.824898
C -1.113556 -0.646502 -1.022631
Zn -0.395251 -1.403039 0.748110
O -1.798601 -0.870098 2.293640
C -1.083501 2.335220 -0.420350
C -3.991288 -1.649240 -0.914956
H -1.200847 3.044334 0.419607
H -0.945383 2.921052 -1.352226
H -0.189106 1.718161 -0.238949
H -4.494306 -1.920067 0.035652
H -3.119699 -2.306046 -1.052879
H -4.707098 -1.810005 -1.743546
C -1.548739 0.290525 3.151794
C -2.585313 -1.870668 3.015833
C -2.429484 0.080594 4.384237
H -1.798000 1.198395 2.572328
H -0.469378 0.306310 3.394234
C -2.507057 -1.454458 4.482629
H -2.150433 -2.859716 2.789805
H -3.625623 -1.831161 2.632231
H -1.591144 -1.862683 4.951788
H -3.379607 -1.806783 5.061205
H -3.437965 0.509321 4.224165
H -1.994931 0.551921 5.283710
Cl 1.531581 -0.389624 1.517071
Cl -0.375950 -3.701490 0.901704
C -0.090057 -0.293871 -2.091324
C -0.972988 -1.510420 -2.265191
H -1.750352 -1.448942 -3.042007
H -0.530310 -2.509808 -2.149254
H 0.973669 -0.435696 -1.853624
H -0.291656 0.561941 -2.753119
H -3.802380 2.696848 -1.299383
H -3.741074 2.658691 0.491691
H -4.888192 0.531768 0.579894
H -5.358532 0.860755 -1.118590

[LiCl(THF)3]:
41
Energy = 0.00 kcal/mol
Li -1.269852 -1.245147 1.019936

O -0.084455 0.285768 1.458545
C 1.132238 0.497282 0.681702
C 0.087207 0.853003 2.776727
C 2.196048 1.021066 1.669486
H 0.916904 1.222989 -0.127417
H 1.390207 -0.477078 0.226029
C 1.584705 0.731158 3.056667
H -0.560440 0.284079 3.467075
H -0.238369 1.916655 2.771556
H 1.826626 -0.297342 3.388160
H 1.930746 1.436471 3.833720
H 2.347715 2.109148 1.536885
H 3.172267 0.524890 1.524307
Cl -0.206984 -2.796026 -0.322503
O -1.816369 -2.028582 2.787345
H -2.254885 1.422031 0.306148
H -3.891525 1.881794 -1.879822
H -4.039515 1.232739 0.490906
C -3.143068 0.880529 -0.064776
H -2.378807 0.942899 -2.098747
C -3.354724 0.964756 -1.576105
O -2.932642 -0.526471 0.199315
C -4.158061 -0.321262 -1.858014
H -5.238671 -0.140482 -1.701342
H -4.022760 -0.691838 -2.889740
C -3.618840 -1.319308 -0.814484
H -2.873276 -2.025400 -1.227323
H -4.428897 -1.896840 -0.326206
C -0.880828 -2.935377 3.419145
C -3.119637 -2.631863 2.937791
C -2.889655 -4.130141 2.690964
H -3.796007 -2.142044 2.216394
H -3.492757 -2.442935 3.968614
C -1.428947 -4.359859 3.172814
H -2.979555 -4.354984 1.612665
H -3.622449 -4.753943 3.233888
H -0.835079 -2.700142 4.503697
H 0.109111 -2.754555 2.966139
H -0.836451 -4.880053 2.399873
H -1.389317 -4.962964 4.098257

[LiCl(THF)2(NHO1)]:
48
Energy = 0.00 kcal/mol
C -3.817733 1.822295 0.366624
C -4.454470 0.460435 0.663232

N -2.516504 1.425754 -0.173523
N -3.835831 -0.379732 -0.369273
C -2.590137 0.142263 -0.693437
C -1.587770 -0.516705 -1.383419
H -0.723520 0.038121 -1.763791
Li -0.473862 -1.114405 0.636765
O 0.887907 0.312071 0.960988
H -1.783900 -1.507647 -1.805562
C -1.594338 2.418198 -0.680524
C -4.086974 -1.808550 -0.372322
H -1.614096 3.300571 -0.016540
H -1.847902 2.749815 -1.713725
H -0.564890 2.017240 -0.678863
H -5.160681 -1.980929 -0.178663
H -3.490654 -2.332041 0.407240
H -3.849724 -2.236479 -1.363793
C 2.214059 0.300898 0.364902
C 0.931239 1.002849 2.234606
C 3.163625 0.932503 1.401770
H 2.187057 0.867193 -0.587343
H 2.465733 -0.753097 0.138972
C 2.369355 0.828712 2.719454
H 0.160712 0.537870 2.875937
H 0.685173 2.076240 2.080226
H 2.498186 -0.169837 3.180717
H 2.662719 1.593705 3.460631
H 3.358392 1.993958 1.155374
H 4.137172 0.411688 1.442235
O 0.633115 -2.732394 0.217467
Cl -1.711308 -1.519603 2.566687
H -4.411841 2.392062 -0.386010
H -3.701024 2.451578 1.268439
H -4.176203 0.100392 1.678915
H -5.555472 0.462595 0.565159
C 0.436966 -3.612510 -0.923219
C 1.071014 -3.504259 1.368586
C 0.846297 -5.017705 -0.456958
H 1.045631 -3.238463 -1.768980
H -0.632032 -3.566917 -1.211566
C 0.647692 -4.940909 1.068696
H 1.909938 -5.211987 -0.695591
H 0.239168 -5.806293 -0.936806
H 0.582428 -3.066440 2.258478
H 2.175055 -3.418314 1.463373
H 1.251154 -5.682699 1.622012
H -0.417095 -5.087033 1.336186

[LiCl(THF)2(NHO2)]:
51
Energy = 0.00 kcal/mol
N -2.714799 1.479865 -0.439159
N -3.884834 -0.552010 -0.470285
C -2.711573 0.133980 -0.754678
C -1.607192 -0.484194 -1.346612
H -0.823582 0.118663 -1.816955
Li -0.508140 -0.988531 0.676179
O 0.730879 0.511005 1.163489
H -1.670852 -1.532189 -1.651723
C -1.583608 2.313195 -0.798438
C -4.019654 -1.933725 -0.896775
H -1.726560 3.316488 -0.363579
H -1.472877 2.424849 -1.900134
H -0.642716 1.888949 -0.395212
H -5.079958 -2.228473 -0.796350
H -3.406181 -2.628540 -0.280113
H -3.729732 -2.042454 -1.957655
C 2.015555 0.715500 0.518292
C 0.760776 1.082529 2.493233
C 2.964619 1.276824 1.598462
H 1.887360 1.414083 -0.333075
H 2.340061 -0.264487 0.119343
C 2.224233 0.981647 2.919745
H 0.050170 0.499282 3.105816
H 0.426596 2.142568 2.448920
H 2.445606 -0.042439 3.278362
H 2.482197 1.693132 3.724801
H 3.095769 2.367983 1.468863
H 3.964894 0.810524 1.553113
O 0.831675 -2.415795 0.255313
Cl -1.767221 -1.584888 2.529619
C 1.519584 -2.998857 1.398276
C 0.839348 -3.341773 -0.859044
C 1.822214 -4.450243 1.009923
H 0.857533 -2.898366 2.278642
H 2.449314 -2.418361 1.574277
C 1.922139 -4.370316 -0.525006
H 0.986070 -5.111981 1.308273
H 2.745260 -4.826551 1.486768
H 1.036102 -2.765708 -1.782058
H -0.160464 -3.818881 -0.941196
H 1.748968 -5.340576 -1.024382
H 2.918346 -3.995969 -0.832769
C -4.760413 -0.199318 0.661791
C -4.442736 1.181945 1.233343
H -4.654405 -0.969772 1.455082

H -5.808693 -0.238516 0.296711
C -3.925119 2.079213 0.110839
H -3.654976 1.098114 2.006089
H -5.345754 1.611944 1.705518
H -3.669045 3.083930 0.490824
H -4.708070 2.210667 -0.673075

[LiCl(THF)2(NHO3)]:
52
Energy = 0.00 kcal/mol
C -3.738446 1.664366 0.673305
C -4.473609 0.504505 0.641227
N -2.597272 1.449843 -0.128914
N -3.784077 -0.400992 -0.188048
C -2.610398 0.166120 -0.641127
C -1.607078 -0.457945 -1.393919
H -0.840893 0.170735 -1.864390
Li -0.363348 -1.248295 0.413055
O 0.938677 0.185907 0.955012
H -1.857059 -1.394258 -1.907844
C -1.529694 2.388399 -0.400483
C -4.124192 -1.784339 -0.442085
H -1.702633 3.319975 0.160720
H -1.480200 2.630123 -1.481593
H -0.555338 1.959639 -0.093468
H -5.106934 -2.011972 -0.000789
H -3.363830 -2.450050 0.014888
H -4.174179 -1.976824 -1.532358
C 2.222093 0.476414 0.337127
C 0.892947 0.776852 2.277399
C 3.078929 1.169130 1.416808
H 2.059268 1.117321 -0.552306
H 2.655845 -0.484258 -0.001394
C 2.350249 0.818164 2.730220
H 0.222479 0.144546 2.886201
H 0.466677 1.802305 2.208097
H 2.661371 -0.177281 3.102633
H 2.525248 1.557915 3.532170
H 3.084305 2.265507 1.263567
H 4.127451 0.822040 1.399740
O 0.806037 -2.820804 -0.116539
Cl -1.552609 -1.991451 2.264578
C 0.103264 -3.924333 -0.757807
C 1.616809 -3.327481 0.979615
C 0.655687 -5.207827 -0.125893
H 0.275117 -3.857825 -1.848704
H -0.980548 -3.804967 -0.554775

C 1.081514 -4.728474 1.274023
H 1.531406 -5.578809 -0.693753
H -0.099288 -6.014386 -0.096997
H 1.509207 -2.628387 1.828732
H 2.678981 -3.352281 0.655312
H 1.838683 -5.380304 1.746341
H 0.203401 -4.655814 1.943986
C -3.989249 2.951685 1.384177
C -5.752463 0.146903 1.320080
H -4.086230 3.809926 0.686767
H -3.178781 3.200768 2.100691
H -4.928312 2.884897 1.960075
H -6.553360 -0.118556 0.598845
H -6.113719 0.999918 1.919522
H -5.627157 -0.715649 2.006943

[LiCl(THF)2(NHO4)]:
54
Energy = 0.00 kcal/mol
C -3.383462 1.691341 0.607517
C -4.160174 0.387770 0.705523
N -2.218863 1.331164 -0.218075
N -3.805413 -0.298411 -0.552012
C -2.523039 0.152845 -0.949129
C -1.704454 -0.440558 -1.891161
C -0.267156 -0.017495 -2.119168
Li -0.474731 -1.179046 0.637980
O 0.906071 0.141730 1.133045
C -2.232002 -1.427155 -2.917713
C -1.657602 2.492816 -0.904980
C -4.121158 -1.726712 -0.514142
H -1.472232 3.285581 -0.157184
H -2.361563 2.895320 -1.670793
H -0.700778 2.266519 -1.395260
H -5.127309 -1.841929 -0.072285
H -3.396192 -2.282520 0.121523
H -4.148412 -2.173405 -1.518742
C 2.300115 0.232461 0.729280
C 0.682562 0.950129 2.318837
C 2.994330 1.123481 1.774038
H 2.352413 0.649787 -0.295125
H 2.715505 -0.793322 0.707870
C 2.051273 1.035317 2.989871
H -0.098111 0.439994 2.911456
H 0.315126 1.952096 2.010842
H 2.247115 0.115520 3.574829

H 2.138008 1.903104 3.668398
H 3.057482 2.167808 1.412773
H 4.020149 0.777302 1.993776
O 0.533721 -2.772502 0.024538
Cl -1.707700 -1.834361 2.461676
H -3.986909 2.474633 0.091470
H -3.074239 2.090660 1.591479
H -3.811882 -0.215402 1.573198
H -5.253169 0.537017 0.781741
C -0.164687 -3.844480 -0.669114
C 1.390324 -3.330446 1.060444
C 0.466330 -5.149793 -0.173435
H -0.050062 -3.683612 -1.757109
H -1.240537 -3.783562 -0.406069
C 0.923732 -4.773663 1.247913
H 1.336657 -5.424396 -0.800879
H -0.249193 -5.991364 -0.191988
H 1.274358 -2.709642 1.967382
H 2.443369 -3.280595 0.711365
H 1.725916 -5.427586 1.634734
H 0.070725 -4.805326 1.952812
H 0.350383 -0.908042 -2.355717
H 0.190511 0.461680 -1.236347
H -0.143307 0.679030 -2.979972
H -1.672399 -1.293546 -3.865269
H -3.299008 -1.260759 -3.156371
H -2.115276 -2.496424 -2.637177

[LiCl(THF)2(NHO5)]:
57
Energy = 0.00 kcal/mol
N -3.017873 1.467183 -0.342322
N -3.840594 -0.659981 -0.945903
C -2.773559 0.238298 -0.997239
C -1.610580 0.013491 -1.725558
C -0.785614 1.161484 -2.266557
Li -0.436673 -1.033458 0.632756
O 1.150420 0.136379 0.647517
C -1.191606 -1.357760 -2.209128
C -1.977934 2.060742 0.498775
C -4.208350 -1.475441 -2.089246
H -2.073850 1.737392 1.558536
H -2.039113 3.165514 0.454635
H -0.981022 1.756194 0.149607
H -5.307805 -1.426807 -2.238182
H -3.941507 -2.549696 -1.977410

H -3.715634 -1.087229 -2.995942
C 2.364243 -0.079926 -0.127365
C 1.456784 0.928937 1.826107
C 3.510494 0.562607 0.674468
H 2.235935 0.374896 -1.128510
H 2.490813 -1.172607 -0.252448
C 2.933973 0.657649 2.100976
H 0.762325 0.604953 2.621712
H 1.278631 2.002246 1.600620
H 3.054168 -0.302322 2.639960
H 3.404737 1.454504 2.704174
H 3.735788 1.573693 0.284918
H 4.437936 -0.035046 0.621312
O 0.359255 -2.870917 0.461527
Cl -1.580213 -1.071859 2.616088
C -0.476000 -4.018219 0.135309
C 1.219204 -3.195076 1.591878
C 0.070199 -5.187119 0.960734
H -0.421388 -4.185418 -0.956146
H -1.522392 -3.777162 0.414723
C 0.641538 -4.473497 2.198829
H 0.874442 -5.710721 0.407862
H -0.714819 -5.924691 1.205990
H 1.203756 -2.336300 2.287040
H 2.252144 -3.348089 1.215098
H 1.405574 -5.068322 2.730938
H -0.167955 -4.219799 2.909960
C -4.682133 -0.823324 0.245794
C -4.687865 0.442905 1.117535
H -4.361211 -1.708005 0.840313
H -5.709892 -1.033086 -0.116624
C -4.370918 1.631107 0.203788
H -3.904742 0.368456 1.896771
H -5.664407 0.554900 1.627183
H -4.408633 2.592725 0.747020
H -5.104860 1.692534 -0.625977
H -0.561546 0.993215 -3.342568
H 0.200128 1.285283 -1.765392
H -1.319505 2.126636 -2.185727
H -0.103881 -1.522310 -2.048946
H -1.350122 -1.504173 -3.302751
H -1.724985 -2.173139 -1.691452

[LiCl(THF)2(NHO6)]:
58
Energy = 0.00 kcal/mol
C -3.415738 1.655444 0.720391

C	-4.194855	0.533759	0.805646
N	-2.423865	1.406321	-0.257016
N	-3.687898	-0.409548	-0.111462
C	-2.553283	0.104001	-0.755323
C	-1.710595	-0.574240	-1.654202
C	-0.369220	-0.020393	-2.106308
Li	-0.517243	-1.342052	0.450516
O	0.611220	0.192058	1.075983
C	-2.248112	-1.597704	-2.640997
C	-1.665085	2.469440	-0.895475
C	-4.063844	-1.809415	-0.105510
H	-2.167657	3.434006	-0.714180
H	-1.629540	2.303227	-1.985221
H	-0.628422	2.545745	-0.513542
H	-4.599028	-2.039661	0.828209
H	-3.150574	-2.432660	-0.129566
H	-4.714191	-2.077565	-0.961680
C	1.982564	0.510763	0.763046
C	0.320048	0.856597	2.325186
C	2.726668	0.529951	2.116369
H	2.019861	1.508292	0.273654
H	2.348831	-0.246448	0.049021
C	1.591614	0.680679	3.171480
H	-0.579856	0.373703	2.742066
H	0.115719	1.930559	2.121663
H	1.510340	-0.229535	3.792919
H	1.752741	1.538725	3.848649
H	3.450162	1.364058	2.157473
H	3.291913	-0.406760	2.271298
O	0.783963	-2.742038	-0.136392
Cl	-1.559082	-2.201469	2.333594
C	0.232858	-3.908649	-0.809370
C	1.704632	-3.164792	0.910186
C	1.070239	-5.100412	-0.337983
H	0.284314	-3.732609	-1.899796
H	-0.830477	-4.013384	-0.510007
C	1.478493	-4.667654	1.082088
H	1.964046	-5.226388	-0.979858
H	0.497417	-6.044830	-0.356950
H	1.473194	-2.581019	1.819716
H	2.739199	-2.938948	0.577354
H	2.379614	-5.188061	1.453235
H	0.650064	-4.845172	1.794452
C	-3.501488	2.948422	1.461249
C	-5.357637	0.233794	1.690956
H	-3.736587	3.810493	0.801853
H	-2.552147	3.188330	1.982810
H	-4.297748	2.894254	2.223458

H -6.249293 -0.079731 1.110096
H -5.633901 1.130443 2.271756
H -5.135028 -0.577154 2.415444
H 0.346525 -0.856616 -2.259947
H 0.099577 0.661411 -1.379804
H -0.417603 0.510243 -3.087383
H -1.583502 -2.482977 -2.731724
H -2.298413 -1.153880 -3.664688
H -3.260921 -1.967263 -2.417639

[LiCl(THF)2(NHO7)]:
58
Energy = 0.00 kcal/mol
C -3.454373 1.841799 0.556100
C -4.324616 0.724568 0.619659
N -2.424296 1.514869 -0.325356
N -3.794451 -0.265026 -0.202238
C -2.600965 0.195861 -0.805449
C -1.791157 -0.503018 -1.689303
C -0.406278 -0.046636 -2.112021
Li -0.468759 -1.350607 0.545319
O 0.609272 0.195533 1.120309
C -2.322723 -1.624542 -2.563668
C -1.585895 2.532876 -0.936613
C -4.213486 -1.653788 -0.107627
H -2.072141 3.515432 -0.805303
H -1.486853 2.342336 -2.018148
H -0.577119 2.581414 -0.483825
H -4.728013 -1.800210 0.856982
H -3.325269 -2.308137 -0.108706
H -4.900255 -1.946700 -0.925421
C 1.979679 0.519981 0.801201
C 0.320364 0.856968 2.374692
C 2.728589 0.551024 2.151409
H 2.008248 1.514197 0.305746
H 2.347242 -0.239973 0.090706
C 1.597048 0.684313 3.212543
H -0.576461 0.370936 2.795086
H 0.109719 1.929550 2.173686
H 1.526634 -0.230284 3.829008
H 1.754441 1.539060 3.894672
H 3.438195 1.396981 2.188428
H 3.310582 -0.375848 2.303805
O 0.790880 -2.745759 -0.102187
Cl -1.636230 -2.217976 2.291471
C 0.239274 -3.911738 -0.779716

C 1.738089 -3.173489 0.920253
C 1.083052 -5.104024 -0.321526
H 0.285748 -3.729671 -1.869380
H -0.821055 -4.021023 -0.472083
C 1.509355 -4.675407 1.094217
H 1.968647 -5.228109 -0.974805
H 0.509477 -6.047867 -0.336595
H 1.534821 -2.590166 1.836447
H 2.764385 -2.951639 0.561319
H 2.413700 -5.198191 1.453689
H 0.689226 -4.851607 1.816244
C -3.719015 3.010801 1.278407
C -5.480040 0.751343 1.409739
H 0.265734 -0.926918 -2.194876
H 0.071483 0.650104 -1.406178
H -0.399748 0.427854 -3.119359
H -1.779472 -2.582619 -2.424573
H -2.178945 -1.349081 -3.632530
H -3.397256 -1.826502 -2.437527
C -4.888188 3.040270 2.075438
C -5.747718 1.930545 2.143813
H -6.647187 1.976932 2.774019
H -6.164543 -0.106071 1.453949
H -3.044673 3.876328 1.239033
H -5.122383 3.946934 2.651074

[LiCl(THF)2(NHO8)]:
52
Energy = 0.00 kcal/mol
C -3.718749 1.829427 0.593082
C -4.431013 0.477774 0.716807
N -2.449794 1.440773 -0.021517
N -3.830759 -0.283844 -0.389168
C -2.568423 0.222443 -0.656048
C -1.561670 -0.446756 -1.350109
C -0.661589 -0.013235 -2.464009
Li -0.494203 -1.204845 0.549135
O 0.841268 0.183288 1.080813
C -1.611588 -1.198836 -2.640273
C -1.445932 2.434046 -0.332326
C -4.126357 -1.704624 -0.478294
H -1.292365 3.083337 0.549371
H -1.737335 3.079831 -1.190422
H -0.493533 1.933323 -0.567889
H -5.221613 -1.844074 -0.431809
H -3.648876 -2.262558 0.356854

H	-3.762261	-2.107244	-1.437188
C	2.157157	0.374444	0.501847
C	0.783372	0.842582	2.368442
C	3.042790	0.983663	1.611941
H	2.075753	1.038752	-0.381037
H	2.513724	-0.617466	0.159609
C	2.214452	0.768902	2.896643
H	0.029015	0.302102	2.966980
H	0.458046	1.897258	2.230775
H	2.407315	-0.231204	3.330654
H	2.420818	1.528340	3.672427
H	3.196547	2.064796	1.432278
H	4.037534	0.506589	1.658590
O	0.667708	-2.789516	0.108740
Cl	-1.745892	-1.793261	2.435130
H	-4.280225	2.532109	-0.065115
H	-3.555673	2.324635	1.568684
H	-4.194744	-0.012385	1.687193
H	-5.527805	0.547803	0.602313
C	0.054355	-3.897699	-0.615606
C	1.424763	-3.307450	1.241960
C	0.560759	-5.182964	0.050921
H	0.345739	-3.815187	-1.678639
H	-1.044237	-3.791123	-0.535156
C	0.882563	-4.715204	1.482312
H	1.476338	-5.548901	-0.452056
H	-0.193579	-5.989479	0.017016
H	1.263998	-2.620308	2.090672
H	2.501038	-3.321093	0.972873
H	1.613099	-5.365689	1.997159
H	-0.038481	-4.661124	2.093481
H	-2.464399	-1.047127	-3.323270
H	-1.174938	-2.208901	-2.705006
H	0.418557	-0.228273	-2.394772
H	-0.888118	0.901075	-3.037166

Lactones

BBL:
12
Energy = 0.00 kcal/mol
C -0.856187 0.032478 0.578922
C -1.929894 -0.823502 -0.097910
C -0.761596 -1.793722 -0.377481
H -2.402673 -0.344989 -0.974352
H -2.710941 -1.194014 0.591547
O 0.168774 -0.857201 0.319346
O -0.783735 1.089068 1.146862
C -0.775238 -3.160162 0.271270
H -0.462892 -1.843408 -1.442117
H 0.225672 -3.627715 0.216957
H -1.492960 -3.818568 -0.256207
H -1.075384 -3.090579 1.334044

GBL:
12
Energy = 0.00 kcal/mol
C -2.142963 0.566821 -0.115613
C -3.233595 -0.472834 0.149713
C -2.541270 -1.814874 -0.123963
H -4.117028 -0.263367 -0.477969
H -3.548943 -0.365816 1.208303
C -1.061655 -1.477116 0.142830
H -2.682089 -2.113217 -1.180420
H -2.898940 -2.643443 0.513485
O -0.922962 -0.063773 -0.139764
H -0.350986 -2.016756 -0.507461
H -0.776962 -1.646571 1.201802
O -2.256022 1.759671 -0.278220

VL:
15
Energy = 0.00 kcal/mol
C -3.298980 0.301462 -1.551945
C -1.787729 0.091175 -1.381939
C -1.508237 -1.401547 -1.189208
H -1.411725 0.689041 -0.529518
H -1.249847 0.452133 -2.283405
C -2.023847 -2.167794 -2.399711
H -2.004321 -1.772209 -0.268190
H -0.424590 -1.603917 -1.079155

O -3.416101 -1.900206 -2.711078
H -1.971132 -3.262442 -2.256174
H -1.431220 -1.912994 -3.304078
C -4.021455 -0.706839 -2.442456
H -3.549576 1.305123 -1.942222
H -3.807434 0.226112 -0.566796
O -5.141367 -0.511696 -2.872420

CL:
18
Energy = 0.00 kcal/mol
C -2.956529 1.248218 -0.230801
C -3.055764 -0.194675 0.317384
C -1.887097 -1.105253 -0.086376
H -3.999335 -0.639373 -0.054811
H -3.148446 -0.157977 1.422549
C -0.503808 -0.607011 0.355389
H -1.888537 -1.218403 -1.192155
H -2.057413 -2.120202 0.324980
C -0.123361 0.778096 -0.172107
H 0.268072 -1.321864 0.002808
H -0.426361 -0.588256 1.462565
O -0.693758 1.871883 0.581712
H -0.380861 0.886666 -1.247060
H 0.964089 0.942600 -0.073482
C -2.030040 2.154290 0.570124
H -3.942894 1.741202 -0.195840
H -2.637945 1.224451 -1.294956
O -2.424914 3.105865 1.216078

Tetrahedral [$MgCl_2$(THF)(Lac)] complexes

[$MgCl_2$(THF)(BBL)] bidentate:
28
Energy = 0.00 kcal/mol
Mg -0.251350 2.440743 -0.561283
O -1.887526 1.771921 0.525715
C -2.957613 2.665611 0.941055
C -4.161423 2.181131 0.136596
H -2.621817 3.692171 0.710393
H -3.110489 2.548417 2.035395
C -3.969516 0.643840 0.109747
H -4.117362 2.604493 -0.884549
H -5.121175 2.483935 0.592926
C -2.455523 0.434856 0.351345
H -4.291770 0.206816 -0.852110
H -4.559582 0.160283 0.910566
H -1.911319 -0.023929 -0.493502
H -2.255884 -0.144983 1.274148
Cl -0.652143 4.699916 -0.800039
Cl 0.227651 0.689442 -1.980302
C 2.430162 2.638762 0.860018
O 3.114168 3.110177 -0.198198
C 4.390515 3.157792 0.603455
C 3.600393 2.618896 1.821889
H 3.494849 3.311304 2.677125
H 3.870367 1.610990 2.184376
C 4.964032 4.552312 0.651921
H 5.083954 2.423376 0.155145
H 5.868089 4.555407 1.291882
H 4.233629 5.271916 1.066213
H 5.257105 4.887394 -0.359867
O 1.245397 2.348776 0.902479

[$MgCl_2$(THF)(BBL)] carbonyl:
28
Energy = 0.00 kcal/mol
Mg -0.264420 2.451919 -0.836846
O -1.832450 1.839537 0.348645
C -2.903449 2.732175 0.765042
C -4.140947 2.145783 0.090534
H -2.621559 3.746477 0.431209

H -2.976495 2.703240 1.873125
C -3.896796 0.618725 0.179645
H -4.182380 2.479816 -0.963360
H -5.077449 2.457940 0.586883
C -2.361810 0.474535 0.301078
H -4.286065 0.087967 -0.707185
H -4.396762 0.192728 1.069335
H -1.875926 -0.015810 -0.561670
H -2.059650 -0.041258 1.233301
Cl -0.646525 4.707519 -1.237985
Cl 0.170370 0.705831 -2.264664
C 2.313373 3.297787 0.367215
O 3.304297 3.438440 1.268884
C 3.993838 4.456774 0.386930
C 2.856378 4.279063 -0.645198
H 3.138956 3.819370 -1.610376
H 2.175370 5.130934 -0.828982
C 5.393673 4.025079 0.026131
H 3.966615 5.421234 0.925942
H 5.836517 4.767586 -0.665989
H 5.391151 3.038343 -0.473563
H 6.033712 3.969327 0.925883
O 1.335632 2.572960 0.491040

[$MgCl_2$(THF)(BBL)] endocyclic:
28
Energy = 0.00 kcal/mol
Mg -0.318716 2.768252 -0.584595
O -2.058811 1.932375 0.066445
C -3.135471 2.669132 0.725895
C -4.395247 1.884845 0.372631
H -3.098010 3.707005 0.351213
H -2.939976 2.670741 1.817772
C -3.885422 0.429990 0.373810
H -4.760900 2.174831 -0.631576
H -5.209725 2.055582 1.099213
C -2.471442 0.548361 -0.207190
H -4.518743 -0.248303 -0.225388
H -3.848720 0.035775 1.407614
H -2.436819 0.406969 -1.303351
H -1.735462 -0.124180 0.267796
Cl -0.485911 5.031964 -0.287055
Cl 0.566309 1.410087 -2.220218
C 1.359917 0.868123 1.388966
O 1.096208 2.169422 0.927938
C 2.576510 2.413649 0.683106
C 2.863426 0.991836 1.210171

H 3.437496 0.923917 2.152166
H 3.262762 0.275298 0.470216
C 3.066795 3.614785 1.450988
H 2.705791 2.507447 -0.410843
H 2.903773 3.493449 2.538323
H 2.546338 4.528193 1.108850
H 4.151337 3.742730 1.268252
O 0.547444 0.066520 1.749754

[$MgCl_2$(THF)(GBL)] bidentate:
28
Energy = 0.00 kcal/mol
Mg -0.842021 2.553824 -0.883949
O -2.459159 1.905395 0.247632
C -3.399727 2.820927 0.872954
C -4.692948 2.648628 0.064718
H -2.949380 3.827528 0.821450
H -3.527048 2.512075 1.932206
C -4.611788 1.192155 -0.482849
H -4.718949 3.377591 -0.765635
H -5.587578 2.819124 0.690665
C -3.209355 0.699723 -0.072701
H -4.723542 1.177257 -1.581671
H -5.397918 0.542059 -0.057485
H -2.651754 0.182460 -0.872765
H -3.242830 0.068227 0.839375
Cl -0.947153 4.853538 -0.696632
Cl -0.782213 1.049554 -2.632931
C 1.931133 2.229338 0.173773
O 2.364977 2.778428 -0.963278
C 3.810111 2.981746 -0.932876
C 4.321695 2.079685 0.206706
H 3.981743 4.059325 -0.747428
H 4.190177 2.718369 -1.933890
C 3.085336 1.948313 1.113607
H 5.196648 2.517353 0.718274
H 4.617523 1.090373 -0.189576
H 3.053943 2.715271 1.915286
H 2.957406 0.964837 1.598188
O 0.735979 2.019143 0.374384

[$MgCl_2$(THF)(GBL)] carbonyl:
28
Energy = 0.00 kcal/mol
Mg -0.529586 2.014013 -0.848950

O -2.274240 1.793266 0.218657
C -3.153325 2.912442 0.526362
C -4.451746 2.556135 -0.191224
H -2.646952 3.824451 0.162974
H -3.285066 2.966377 1.627926
C -4.542773 1.024796 0.008509
H -4.367102 2.812583 -1.264394
H -5.323603 3.093097 0.223945
C -3.074467 0.566169 0.153036
H -5.044413 0.524262 -0.838561
H -5.115185 0.785833 0.924371
H -2.688917 -0.009843 -0.707803
H -2.898064 -0.007809 1.083107
Cl -0.347974 4.281110 -1.308332
Cl -0.400482 0.204368 -2.269606
C 2.176064 1.948664 0.352749
O 3.066648 1.693247 1.318819
C 4.422375 1.993286 0.859479
C 4.248888 2.901791 -0.371405
H 4.904288 1.025668 0.619174
H 4.949351 2.461390 1.707846
C 2.845286 2.524559 -0.873688
H 5.041587 2.734247 -1.121231
H 4.278813 3.966839 -0.075210
H 2.850477 1.733769 -1.653634
H 2.232688 3.361162 -1.264824
O 0.980573 1.709971 0.529012

[$MgCl_2$(THF)(GBL)] endocyclic:
28
Energy = 0.00 kcal/mol
Mg -1.424992 2.886904 -1.863616
O -2.692010 1.894400 -0.613854
C -3.100589 2.418331 0.694140
C -4.434059 1.732125 0.979081
H -3.146924 3.518052 0.602298
H -2.315384 2.144725 1.425102
C -4.247611 0.355779 0.311309
H -5.266477 2.287602 0.505101
H -4.639875 1.661156 2.062083
C -3.460099 0.694951 -0.956290
H -5.204143 -0.145499 0.078372
H -3.660044 -0.316018 0.966717
H -4.116793 0.945830 -1.811338
H -2.748950 -0.086497 -1.276652
Cl -1.562291 5.120294 -1.356104

Cl -1.342361 1.642292 -3.794974
C 1.089305 2.348799 0.061757
O 0.615505 2.510154 -1.236236
C 1.703352 2.832469 -2.170681
C 2.973048 2.361974 -1.447585
H 1.675236 3.927026 -2.332575
H 1.469233 2.307551 -3.112025
C 2.598274 2.512118 0.035608
H 3.853646 2.959020 -1.741709
H 3.181192 1.302564 -1.689150
H 2.822027 3.526494 0.426901
H 3.068885 1.784632 0.718743
O 0.335033 2.121457 0.974390

$[MgCl_2(THF)(VL)]$ bidentate:
31
Energy = 0.00 kcal/mol
O -2.637330 1.732122 0.497607
C -4.006835 2.144028 0.759553
C -4.852342 1.355957 -0.254495
H -4.040299 3.240457 0.636327
H -4.252518 1.868397 1.806865
C -3.978288 0.114229 -0.606788
H -5.040514 1.970363 -1.153288
H -5.832390 1.076245 0.172807
C -2.687529 0.307651 0.209192
H -3.749615 0.089072 -1.687378
H -4.475033 -0.838138 -0.346527
H -1.757917 0.058343 -0.331250
H -2.724651 -0.235083 1.177161
Cl -2.401403 4.963213 -0.656523
Cl 0.005487 1.596696 -1.701271
Mg -1.211664 3.005729 -0.326295
C 1.917016 4.306419 2.421630
C 2.897273 5.474648 2.243862
C 3.466378 5.444983 0.821109
H 3.701097 5.409224 3.000244
H 2.373708 6.437040 2.417238
C 2.334767 5.560550 -0.188198
H 4.032388 4.506358 0.650497
H 4.168675 6.283178 0.647767
O 1.246789 4.616992 0.062598
H 2.653077 5.338378 -1.221110
H 1.870780 6.566122 -0.171527
C 0.992773 4.075334 1.248151
H 1.280625 4.405070 3.320373

H 2.467147 3.349157 2.547424
O -0.004409 3.346731 1.341853

[$MgCl_2$(THF)(VL)] carbonyl:
31
Energy = 0.00 kcal/mol
O -2.310602 1.573386 0.389196
C -3.337561 2.508798 -0.046832
C -4.249796 1.655677 -0.923568
H -2.817719 3.331510 -0.569526
H -3.863279 2.899654 0.850129
C -4.264094 0.294893 -0.186056
H -3.807529 1.555895 -1.932863
H -5.258741 2.092952 -1.031404
C -2.928727 0.260813 0.592229
H -4.348739 -0.554486 -0.886919
H -5.120177 0.238411 0.511997
H -2.202367 -0.485260 0.222016
H -3.077151 0.116971 1.680078
Cl -0.235298 3.683293 -1.473859
Cl 0.452610 -0.344306 -0.594603
Mg -0.334525 1.788208 -0.147721
C 2.809472 2.382770 0.461401
C 4.309404 2.516262 0.758129
C 4.527917 3.608142 1.809504
H 4.856466 2.740211 -0.175956
H 4.708299 1.552750 1.135537
C 3.749006 3.260528 3.067837
H 4.200213 4.594571 1.421968
H 5.597262 3.704051 2.081317
O 2.332083 3.001813 2.816353
H 3.746508 4.077060 3.810859
H 4.151252 2.347651 3.551411
C 1.885515 2.562673 1.641182
H 2.545623 1.403784 0.006755
H 2.466725 3.143237 -0.276246
O 0.668100 2.342610 1.556490

[$MgCl_2$(THF)(CL)] bidentate:
34
Energy = 0.00 kcal/mol
Mg -0.905762 1.700609 -0.396762
O 1.835063 2.181822 -0.265044
O -2.735187 1.410906 0.557812
C -3.684000 2.483964 0.806318
C -4.886653 2.167397 -0.100976

H -3.165540 3.429913 0.572694
H -3.958688 2.454665 1.881783
C -4.764169 0.640946 -0.391990
H -4.826757 2.751339 -1.036894
H -5.839310 2.426473 0.395811
C -3.522530 0.198757 0.399552
H -4.612219 0.458748 -1.471079
H -5.661274 0.076909 -0.078393
H -2.881349 -0.533886 -0.120486
H -3.786410 -0.172429 1.412337
C 2.945832 2.922526 -0.864444
C 3.457359 4.082238 -0.021267
H 2.502574 3.281285 -1.808708
H 3.734150 2.183209 -1.104917
C 4.419402 3.665074 1.107324
H 2.576801 4.629890 0.371849
H 3.960461 4.793910 -0.704666
C 1.524446 2.049888 1.020166
Cl -0.960473 3.973530 -0.912616
Cl -0.695747 -0.210560 -1.675535
O 0.376294 1.642494 1.261138
C 2.487141 2.333419 2.154021
C 3.983376 2.371863 1.816498
H 2.261128 1.556755 2.908785
H 2.153188 3.287963 2.614852
H 4.236319 1.488822 1.195454
H 4.559305 2.248137 2.753321
H 4.493527 4.490895 1.843062
H 5.439853 3.524291 0.698551

[$MgCl_2$(THF)(CL)] carbonyl:
34
Energy = 0.00 kcal/mol
Mg -0.597582 2.286334 -0.498812
O 2.838768 1.279019 1.665116
O -2.330015 1.537214 0.336032
C -3.330288 2.395914 0.956545
C -4.590811 2.132739 0.139216
H -2.938746 3.427670 0.906172
H -3.451204 2.090089 2.017502
C -4.498311 0.615235 -0.134499
H -4.553701 2.710420 -0.804208
H -5.511216 2.414272 0.681961
C -2.982851 0.331253 -0.187479
H -5.001445 0.324438 -1.073802
H -4.971118 0.047007 0.689023

H -2.584002 0.178844 -1.207196
H -2.685563 -0.523856 0.449242
C 4.289013 1.339193 1.824922
C 5.070146 1.496486 0.523618
H 4.510809 0.372404 2.308673
H 4.514850 2.142303 2.553884
C 5.210153 2.957933 0.054132
H 4.576268 0.873192 -0.249356
H 6.070601 1.044115 0.670361
C 2.104715 1.966256 0.790912
Cl -0.695690 4.579574 -0.139540
Cl -0.293145 1.113497 -2.463197
O 0.906819 1.647121 0.736456
C 2.641385 3.078682 -0.087270
C 3.941839 3.783731 0.317406
H 1.803789 3.807925 -0.139502
H 2.712841 2.652153 -1.112143
H 3.880750 4.071373 1.386358
H 3.997119 4.740148 -0.236103
H 5.450738 2.970630 -1.027728
H 6.068207 3.437514 0.566110

Trigonal-bipyramidal [$MgCl_2(THF)_2$(Lac)] complexes

[$MgCl_2(THF)_2$(BBL)] bidentate:
41
Energy = 0.00 kcal/mol
Mg 0.341164 1.781919 0.581905
Cl 1.491166 3.716325 1.290463
O 2.134440 0.953885 -0.361121
O -2.110351 4.233514 0.380164
Cl -1.122284 0.872015 -1.025061
C 3.257157 1.736728 -0.842961
C 3.155516 1.661727 -2.375303
H 4.192156 1.260043 -0.475601
H 3.158117 2.743068 -0.403286
C 2.390897 0.330290 -2.638651
H 4.155298 1.678902 -2.846621
H 2.585305 2.524260 -2.765747
C 2.072116 -0.204892 -1.230144
H 2.991993 -0.395220 -3.216991
H 1.453122 0.516631 -3.191558
H 2.836652 -0.935507 -0.886856

H 1.059897 -0.631262 -1.134727
C -3.357715 4.921377 0.790218
C -2.184859 3.392556 1.440816
O 0.575428 0.357554 2.095366
C 1.713782 0.417196 3.006751
C 1.200159 -0.177882 4.318705
H 2.034025 1.473496 3.068872
H 2.534185 -0.185637 2.567423
C 0.150662 -1.196117 3.832418
H 0.723389 0.607359 4.937029
H 2.009674 -0.638294 4.913140
C -0.489335 -0.467837 2.648353
H -0.590532 -1.461022 4.607859
H 0.642186 -2.129935 3.495930
H -1.314385 0.200331 2.964003
H -0.855054 -1.129658 1.843179
O -1.435201 2.476815 1.713212
C -3.453126 4.003696 2.033794
H -4.133939 4.773688 0.018635
H -3.157930 5.992579 0.964925
C -4.660862 3.084292 2.198756
H -3.221518 4.539113 2.975722
H -4.874367 2.531878 1.264803
H -5.558377 3.669042 2.474854
H -4.474852 2.345123 2.999732

[$MgCl_2(THF)_2(BBL)$] carbonyl:
41
Energy = 0.00 kcal/mol
Mg 0.340304 1.460711 1.051466
Cl 1.845853 3.215074 1.530918
O 4.207733 -0.556982 0.260502
O -1.428497 2.624383 1.558427
Cl -0.585303 0.566864 -0.917049
C 3.104625 0.240964 0.212119
C 4.905355 0.301219 -0.734719
C -1.406564 3.874722 2.290722
C -1.969181 4.915805 1.307436
H -0.362637 4.054133 2.596988
H -2.058337 3.765035 3.184873
C -2.835023 4.076070 0.321366
H -1.142897 5.420230 0.774762
H -2.555877 5.691084 1.833292
C -2.677199 2.628770 0.821134
H -2.456325 4.169036 -0.712579
H -3.895839 4.386750 0.320268
H -2.584261 1.868973 0.027354

H -3.493706 2.350608 1.522713
O -0.061797 0.229420 2.702169
C 0.844832 0.164330 3.846034
C 0.689066 -1.251694 4.414103
H 0.558293 0.962699 4.555105
H 1.869610 0.361440 3.478197
C 0.238337 -2.067583 3.187028
H -0.089269 -1.273828 5.201697
H 1.630468 -1.622654 4.856951
C -0.665663 -1.077180 2.457496
H -0.293040 -2.997523 3.458114
H 1.105456 -2.330568 2.552266
H -1.693173 -1.066714 2.877019
H -0.722954 -1.216211 1.362359
O 2.087695 0.079756 0.858574
C 2.931968 1.401931 -2.124581
C 3.670418 1.230318 -0.798833
H 5.123709 -0.285686 -1.644386
H 5.826325 0.714595 -0.288320
H 3.518601 2.050355 -2.802092
H 1.945200 1.869076 -1.956342
H 2.760656 0.429427 -2.622372
H 3.820115 2.207331 -0.297111

[$MgCl_2(THF)_2$(BBL)] endocyclic:
41
Energy = 0.00 kcal/mol
Mg -0.088239 1.437254 0.717584
Cl 1.510619 3.148169 0.568703
O 1.652883 0.041349 -0.141289
O -1.602284 2.624415 1.675416
Cl -1.446972 0.403582 -0.876615
C 2.995519 -0.043605 0.225593
C 2.023710 0.289044 -1.564237
C -1.517321 4.058155 1.891400
C -2.513172 4.658715 0.888337
H -0.464540 4.346279 1.734021
H -1.818608 4.265583 2.940966
C -3.561515 3.526686 0.673002
H -1.997520 4.900707 -0.058525
H -2.964997 5.590417 1.274593
C -3.016015 2.342080 1.493694
H -3.633451 3.252864 -0.394590
H -4.571053 3.817347 1.016642
H -3.090333 1.368436 0.981199
H -3.486584 2.286933 2.498465
O 0.100041 0.238594 2.397462

C 0.904255 0.619669 3.560060
C 1.009339 -0.650677 4.408393
H 0.387688 1.456036 4.064830
H 1.890028 0.961229 3.192479
C 0.901919 -1.772682 3.357666
H 0.171609 -0.710549 5.130628
H 1.955564 -0.685568 4.976667
C -0.119693 -1.204521 2.374465
H 0.572203 -2.735610 3.787594
H 1.875538 -1.920954 2.854796
H -1.163493 -1.398586 2.694310
H 0.003398 -1.544830 1.331711
O 3.426013 -0.242231 1.329753
C 4.428188 1.380745 -1.411797
C 3.519743 0.170426 -1.195605
H 1.683412 1.294877 -1.861288
H 1.582696 -0.495745 -2.199539
H 4.614397 1.530917 -2.491829
H 5.402481 1.224031 -0.913609
H 3.962559 2.294497 -0.999136
H 3.986560 -0.758616 -1.579497

$[MgCl_2(THF)_2(GBL)]$ bidentate:
41
Energy = 0.00 kcal/mol
Mg 0.272867 1.095595 0.957020
Cl 0.394202 3.072431 2.255714
O 2.330669 1.368574 0.260887
O -2.827113 2.234746 0.893591
Cl -0.460679 0.213904 -1.099393
C 2.871861 2.665695 -0.093697
C 2.836366 2.673951 -1.624767
H 3.912701 2.731141 0.293651
H 2.247669 3.422261 0.410688
C 3.106634 1.190739 -1.999759
H 3.580038 3.368735 -2.056163
H 1.833259 2.981797 -1.972739
C 2.907918 0.420284 -0.673516
H 4.132213 1.047203 -2.387970
H 2.395192 0.837434 -2.766031
H 3.872917 0.065916 -0.253432
H 2.200134 -0.420739 -0.760064
C -4.128826 2.873111 1.012499
C -4.997810 1.882377 1.809979
H -4.484782 3.072999 -0.012047
H -3.976434 3.833928 1.541462

C -3.948655 1.110576 2.626330
H -5.532008 1.200016 1.122457
H -5.749774 2.398422 2.432359
C -2.680312 1.263274 1.804712
H -4.175175 0.044066 2.796589
H -3.754759 1.571260 3.617111
O -1.636200 0.634175 1.924962
O 1.041822 -0.493229 2.090550
C 1.850071 -0.212744 3.271831
C 1.404102 -1.248352 4.303252
H 1.664920 0.839857 3.555303
H 2.917942 -0.333119 2.996746
C 1.061193 -2.463071 3.418836
H 0.505305 -0.889977 4.841565
H 2.190157 -1.462075 5.049588
C 0.427517 -1.811838 2.184760
H 0.375226 -3.178164 3.907675
H 1.982735 -3.010978 3.140626
H -0.664768 -1.670912 2.295832
H 0.622663 -2.346875 1.237887

$[MgCl_2(THF)_2(GBL)]$ carbonyl:
41
Energy = 0.00 kcal/mol
Mg 0.160897 1.427227 0.934776
Cl 0.661216 3.430175 2.090165
O 2.161693 1.246970 0.103665
O -3.877129 1.673765 2.419084
Cl -0.889192 1.081704 -1.151976
C 2.904795 2.419071 -0.337792
C 3.126517 2.189954 -1.829592
H 3.864516 2.460472 0.220493
H 2.303246 3.304770 -0.071221
C 3.373458 0.669847 -1.885303
H 3.973036 2.779086 -2.226954
H 2.212376 2.457138 -2.393296
C 2.443620 0.123903 -0.788433
H 4.432187 0.441948 -1.652884
H 3.141170 0.232140 -2.872850
H 2.904785 -0.686814 -0.191729
H 1.471858 -0.216847 -1.194297
C -4.887772 2.664721 2.064813
C -4.106168 3.890280 1.558135
H -5.491912 2.845492 2.970402
H -5.529715 2.213347 1.282769
C -2.790955 3.267379 1.064169

H -3.912581 4.596720 2.387132
H -4.658026 4.432408 0.770481
C -2.701609 1.969277 1.828428
H -1.874976 3.858824 1.256685
H -2.784949 3.005270 -0.015499
O -1.740367 1.217021 1.950267
O 0.714566 -0.263972 2.053888
C 1.665628 -0.068429 3.145695
C 1.166623 -0.976070 4.269406
H 1.673949 1.009451 3.392572
H 2.671029 -0.365223 2.783790
C 0.533615 -2.146157 3.492276
H 0.403373 -0.452025 4.876186
H 1.983122 -1.293599 4.942422
C -0.115783 -1.439118 2.299598
H -0.204214 -2.713719 4.087240
H 1.315352 -2.853236 3.152168
H -1.141348 -1.090136 2.523161
H -0.133395 -2.040139 1.372203

$[MgCl_2(THF)_2(VL)]$ bidentate:
44
Energy = 0.00 kcal/mol
Mg 0.098675 1.594081 0.774999
Cl 1.023241 3.564009 1.717575
O 2.046259 1.066605 -0.116106
O -2.274823 3.572463 0.528130
Cl -1.103761 0.684674 -1.051219
C 3.049756 2.023248 -0.538643
C 2.991100 1.991898 -2.073814
H 4.038192 1.677005 -0.164075
H 2.794843 2.985642 -0.064735
C 2.477786 0.558261 -2.400085
H 3.979024 2.204501 -2.522144
H 2.281327 2.753568 -2.445069
C 2.180972 -0.058513 -1.018670
H 3.224570 -0.040494 -2.953508
H 1.552959 0.597403 -3.002200
H 3.024566 -0.688822 -0.661815
H 1.235419 -0.623944 -0.981098
C -3.196544 4.552222 -0.028421
C -3.841817 2.993056 2.349915
C -2.568203 2.779316 1.556047
O 0.480860 0.037753 2.131868
C 1.570891 0.153784 3.094647
C 1.074554 -0.578988 4.341681
H 1.773417 1.231181 3.237923

H 2.467775 -0.329214 2.656254
C 0.168716 -1.672661 3.743406
H 0.486056 0.106707 4.981747
H 1.905722 -0.984897 4.945968
C -0.500017 -0.942871 2.575623
H -0.569907 -2.068324 4.463664
H 0.777154 -2.522267 3.376111
H -1.412214 -0.398433 2.888182
H -0.747192 -1.587101 1.712830
C -4.232238 5.042087 0.972720
H -3.674078 4.061953 -0.900068
H -2.544414 5.362709 -0.397748
C -4.908082 3.841352 1.642846
H -4.965585 5.671802 0.432391
H -3.747235 5.686677 1.734252
H -3.515304 3.486356 3.290391
H -4.211658 1.993825 2.643866
H -5.428555 3.231054 0.876427
H -5.677257 4.164667 2.369086
O -1.755235 1.905891 1.866870

[$MgCl_2(THF)_2(VL)$] carbonyl:
44
Energy = 0.00 kcal/mol
Mg 0.760590 1.567904 0.961966
Cl 1.926917 3.426477 1.855753
O 2.448533 0.983161 -0.286018
O -2.590360 2.553361 3.465157
Cl -0.819539 1.337083 -0.782160
C 3.385213 1.925963 -0.865751
C 2.961275 2.019058 -2.334555
H 4.410907 1.508643 -0.760220
H 3.304527 2.857693 -0.281406
C 2.428683 0.593776 -2.650212
H 3.800179 2.319043 -2.988792
H 2.153788 2.765675 -2.445410
C 2.285144 -0.076087 -1.263733
H 3.129235 0.025781 -3.289995
H 1.453278 0.638964 -3.165274
H 3.077024 -0.834119 -1.087469
H 1.291904 -0.525599 -1.097371
C -3.603626 3.568297 3.729049
C -1.650675 3.913499 1.610892
C -1.648118 2.714748 2.522849
O 1.109968 -0.237004 1.983153
C 2.031634 -0.186032 3.113374

C 1.248310 -0.783428 4.280016
H 2.331828 0.869587 3.250498
H 2.927640 -0.790321 2.860731
C 0.394583 -1.859541 3.580992
H 0.603186 -0.010908 4.739871
H 1.911452 -1.197256 5.060688
C 0.042511 -1.202299 2.240169
H -0.510097 -2.130907 4.153776
H 0.987236 -2.781949 3.423621
H -0.910367 -0.643033 2.281856
H 0.011282 -1.910295 1.391708
C -3.937559 4.413110 2.509515
H -3.216828 4.198390 4.555946
H -4.471351 2.997476 4.104548
C -2.649523 5.026960 1.953250
H -4.662986 5.192411 2.815174
H -4.434031 3.786003 1.740670
H -1.834456 3.476906 0.602988
H -0.597942 4.265852 1.578668
H -2.211742 5.711788 2.708103
H -2.852149 5.637726 1.053999
O -0.810950 1.813978 2.420426

$[MgCl_2(THF)_2(CL)]$ bidentate:
47
Energy = 0.00 kcal/mol
Mg 0.274627 1.621109 1.047393
Cl 1.449036 3.646203 1.490841
O 1.880225 0.928190 -0.271635
O -1.981463 3.740052 1.213027
Cl -1.392976 0.555393 -0.242862
C 2.766953 1.775290 -1.042583
C 2.328468 1.568455 -2.501380
H 3.809690 1.424421 -0.879777
H 2.660433 2.798592 -0.646630
C 1.712478 0.138056 -2.508295
H 3.179880 1.670014 -3.199214
H 1.569697 2.320948 -2.784109
C 1.761770 -0.299761 -1.031147
H 2.277796 -0.560955 -3.151888
H 0.666292 0.160246 -2.861392
H 2.655740 -0.925079 -0.818082
H 0.844982 -0.808347 -0.690547
C -1.954269 3.154024 2.409719
C -2.783503 4.913428 0.919836
O 0.922585 0.202711 2.462582

C 1.915151 0.589623 3.455661
C 1.272268 0.241110 4.795888
H 2.127753 1.663615 3.301567
H 2.840616 0.002451 3.276929
C 0.491812 -1.043766 4.454787
H 0.582984 1.049244 5.107242
H 2.020350 0.092614 5.595440
C 0.007544 -0.790497 3.018650
H -0.349597 -1.234746 5.144839
H 1.164356 -1.922818 4.491628
H -1.011933 -0.364721 2.977235
H 0.041082 -1.688780 2.375033
C -2.818309 3.679729 3.534358
C -2.378442 5.079095 4.031525
H -2.741601 2.938559 4.347663
H -3.878877 3.709095 3.206937
C -2.834606 6.237412 3.132467
H -1.275125 5.092880 4.142023
H -2.797049 5.224345 5.046250
C -2.333532 6.158156 1.682400
H -2.500332 7.194160 3.580131
H -3.945944 6.271572 3.127261
H -1.226234 6.202501 1.640703
H -2.706071 7.039001 1.121280
H -3.852267 4.674932 1.099008
H -2.641421 5.041917 -0.166028
O -1.187199 2.200421 2.565991

[$MgCl_2(THF)_2(CL)$] carbonyl:
47
Energy = 0.00 kcal/mol
Mg 0.256366 2.012271 1.056024
Cl 1.446157 4.033695 1.343915
O 1.866322 1.134475 -0.153101
O -3.120776 3.211773 3.409126
Cl -1.431013 1.316338 -0.443221
C 2.718411 1.913234 -1.031241
C 2.148196 1.660400 -2.431100
H 3.758565 1.530299 -0.937530
H 2.675075 2.956380 -0.676106
C 1.589456 0.212046 -2.344319
H 2.916917 1.777894 -3.216676
H 1.332116 2.376191 -2.638714
C 1.635021 -0.123851 -0.836642
H 2.200435 -0.503034 -2.925841
H 0.552294 0.165274 -2.718894

H 2.474932 -0.808783 -0.592803
H 0.688014 -0.537436 -0.451880
C -2.129181 3.396932 2.520629
C -4.271460 4.097136 3.468787
O 0.823045 0.557276 2.458452
C 2.116082 0.682995 3.122317
C 1.869536 0.206915 4.553508
H 2.435119 1.737876 3.034991
H 2.843693 0.039234 2.587446
C 0.783365 -0.868502 4.358590
H 1.486762 1.039752 5.174565
H 2.787808 -0.183504 5.027668
C -0.085274 -0.267410 3.249799
H 0.201580 -1.069038 5.276051
H 1.238376 -1.822522 4.027287
H -0.882813 0.390326 3.643262
H -0.537672 -1.015821 2.574111
C -2.149528 4.597128 1.606150
C -3.256202 4.523623 0.526139
H -1.143877 4.648730 1.150858
H -2.272898 5.516140 2.217798
C -4.654151 4.892644 1.040922
H -3.245535 3.510027 0.077307
H -2.973682 5.220743 -0.285765
C -5.150784 4.040013 2.219103
H -5.380473 4.815888 0.207885
H -4.655396 5.959991 1.352327
H -5.257756 2.976901 1.920480
H -6.163138 4.382760 2.515978
H -3.928193 5.129938 3.685089
H -4.819464 3.729816 4.353035
O -1.242440 2.541845 2.505926

$[MgCl_2(THF)_2(CL)]$ endocyclic:
47
Energy = 0.00 kcal/mol
Mg 0.041834 1.455005 0.511523
Cl 0.226531 3.751853 0.879271
O 2.035022 1.199291 -0.228879
O -2.330811 1.739588 1.631070
Cl -1.059373 0.164537 -1.098551
C 2.872108 2.266685 -0.754630
C 3.245712 1.789072 -2.154096
H 3.767003 2.371896 -0.104482
H 2.278060 3.194965 -0.702335
C 3.454918 0.276953 -1.932985

H	4.145903	2.294723	-2.547822
H	2.407312	1.973499	-2.852627
C	2.436920	-0.072818	-0.830002
H	4.488679	0.079091	-1.589707
H	3.285054	-0.317344	-2.848695
H	2.867736	-0.713436	-0.035984
H	1.514458	-0.541466	-1.220099
C	-2.335614	2.291705	2.882836
C	-3.424047	1.963989	0.679050
O	0.503136	0.180369	2.061547
C	1.348045	0.614122	3.176785
C	0.996280	-0.317776	4.341322
H	1.126361	1.675701	3.384122
H	2.400122	0.511132	2.846274
C	0.446356	-1.572483	3.634339
H	0.210068	0.145761	4.964750
H	1.872041	-0.530453	4.980460
C	-0.306323	-0.967306	2.452519
H	-0.214072	-2.176538	4.281911
H	1.270219	-2.222792	3.280111
H	-1.308708	-0.602584	2.748235
H	-0.397512	-1.618518	1.565856
C	-3.384827	3.335465	3.214562
C	-3.325655	4.604972	2.331262
H	-3.213457	3.588623	4.274805
H	-4.394383	2.876271	3.147671
C	-4.029156	4.455806	0.975107
H	-2.265839	4.890415	2.176738
H	-3.800555	5.429962	2.897101
C	-3.432694	3.364635	0.075970
H	-3.991891	5.425559	0.440329
H	-5.106595	4.239609	1.148094
H	-2.392347	3.623826	-0.205172
H	-4.011255	3.304421	-0.868089
H	-4.378589	1.713316	1.184253
H	-3.218554	1.206412	-0.095861
O	-1.481977	1.921583	3.670258

Structures related to the proton transfer reaction

$[MgCl_2(THF)(DVL)BuOH]$...NHO
76
geomtry on BP-86/def2-SVPD level incl. COSMO (ε = 7.58)
O -2.118566 2.399370 -0.409960
C -2.247434 2.702583 -1.821219
C -3.752411 2.926807 -2.031909
H -1.812762 1.854686 -2.374001
H -1.667443 3.625499 -2.038753
C -4.264829 3.390546 -0.635998
H -4.243402 1.984575 -2.335772
H -3.939480 3.675478 -2.822518
C -3.023232 3.303866 0.272423
H -5.065780 2.724073 -0.268941
H -4.667227 4.419306 -0.657642
H -3.210052 2.879888 1.272639
H -2.525926 4.292567 0.371920
Cl -0.470321 -0.236207 -1.412310
Cl -2.399453 0.441879 2.235252
Mg -0.758870 1.032685 0.593844
C 1.309761 4.916525 1.232120
C 2.427919 5.663353 0.493315
C 2.164061 5.598977 -1.013780
H 3.409908 5.202048 0.722487
H 2.474369 6.708315 0.849023
C 2.123466 4.150942 -1.466564
H 2.960670 6.109508 -1.587520
H 1.208790 6.105235 -1.261099
O 1.230002 3.313069 -0.648370
H 3.119234 3.673278 -1.401294
H 1.741201 4.028823 -2.493518
C 0.895671 3.609134 0.602032
H 0.383760 5.530630 1.254098
H 1.548037 4.710309 2.291670
O 0.198405 2.788096 1.219664
O 0.840710 0.001623 1.543042
C 1.688709 0.459534 2.616192
H 0.889654 -1.001305 1.494581
C 3.070116 0.874353 2.112849
H 1.775217 -0.346327 3.372044
H 1.176192 1.312475 3.099303
C 3.972420 1.421884 3.228193
H 2.942072 1.639652 1.319766
H 3.555780 0.005039 1.623557

C 5.325075 1.928369 2.713539
H 4.135511 0.630233 3.988805
H 3.444243 2.244589 3.754820
H 5.884150 1.123545 2.196524
H 5.957205 2.302429 3.541174
H 5.190948 2.758000 1.990904
C 1.040649 -2.973778 1.379399
C 2.011258 -2.819174 0.219049
H 2.985839 -2.393091 0.514074
H 1.568720 -2.128044 -0.526827
H 2.201875 -3.772685 -0.321115
C -0.379407 -3.304534 0.942378
H -0.902027 -2.446132 0.473683
H -1.011444 -3.696151 1.755265
H -0.337934 -4.097571 0.163835
N 2.759542 -3.443475 3.154637
N 0.663457 -3.036845 3.857320
C 2.773465 -3.480487 4.548830
C 1.446971 -3.241999 4.989399
C 3.807271 -3.729883 5.458776
C 3.481838 -3.737173 6.835604
H 4.837204 -3.908987 5.122475
C 2.168118 -3.492821 7.272657
H 4.272845 -3.936453 7.572243
C 1.123670 -3.243530 6.352151
H 1.942054 -3.501834 8.348287
H 0.095719 -3.073970 6.698427
C 3.807430 -4.072455 2.361629
H 4.350238 -4.790254 3.002300
H 4.535560 -3.342354 1.960980
H 3.357403 -4.631103 1.525567
C -0.663905 -2.444395 3.927706
H -0.753641 -1.894214 4.880767
H -1.467316 -3.204673 3.888612
H -0.813592 -1.723874 3.104821
C 1.458344 -3.147682 2.695075

$[MgCl_2(THF)(DVL)BuO]^-$...$NHOH^+$
76
geomtry on BP-86/def2-SVPD level incl. COSMO (ε = 7.58)
O -2.247583 2.313960 -0.375041
C -2.302032 2.556652 -1.797569
C -3.774167 2.906477 -2.083257
H -1.929797 1.643748 -2.290069
H -1.631883 3.410899 -2.038973
C -4.316329 3.410114 -0.712374
H -4.328397 2.011861 -2.420261

H	-3.856295	3.671865	-2.876302
C	-3.103099	3.308207	0.229731
H	-5.139339	2.765335	-0.354449
H	-4.698063	4.446009	-0.762763
H	-3.332985	2.956796	1.249025
H	-2.552130	4.272851	0.283297
Cl	-0.900152	-0.512106	-1.124464
Cl	-2.578793	0.655041	2.465999
Mg	-0.846280	1.042400	0.777601
C	1.197769	5.024584	1.267368
C	2.408582	5.615415	0.532450
C	2.190992	5.480095	-0.976947
H	3.334834	5.081424	0.826704
H	2.541954	6.672261	0.827406
C	2.018033	4.014853	-1.337892
H	3.051882	5.873233	-1.550558
H	1.297691	6.058792	-1.289414
O	0.990695	3.340480	-0.537835
H	2.953804	3.445549	-1.176185
H	1.693876	3.866360	-2.381562
C	0.662714	3.728835	0.698295
H	0.342765	5.731928	1.216797
H	1.382017	4.855628	2.343739
O	-0.127924	3.022743	1.331140
O	0.759958	0.262527	1.573117
C	1.623513	0.679895	2.567664
H	0.797011	-1.650408	1.440681
C	2.998916	1.161429	2.057332
H	1.840579	-0.151925	3.296898
H	1.190855	1.502037	3.199417
C	3.974181	1.526229	3.185572
H	2.848305	2.034407	1.386273
H	3.433725	0.364595	1.416802
C	5.336976	2.028983	2.693785
H	4.123768	0.638611	3.837240
H	3.509288	2.297183	3.836868
H	5.842504	1.264052	2.070796
H	6.011536	2.276619	3.536721
H	5.224168	2.941078	2.073848
C	1.167654	-2.711162	1.346566
C	2.265584	-2.613772	0.274709
H	3.152757	-2.063709	0.636194
H	1.842053	-2.031585	-0.562887
H	2.583365	-3.595661	-0.120625
C	-0.003995	-3.609400	0.883966
H	-0.422843	-3.175672	-0.042404
H	-0.818676	-3.655460	1.629118
H	0.335610	-4.643791	0.680379

N 2.696374 -3.754796 3.159546
N 0.882166 -2.690785 3.832875
C 2.697071 -3.806322 4.556807
C 1.525560 -3.139603 4.983333
C 3.601504 -4.362887 5.474226
C 3.281636 -4.234200 6.833906
H 4.520153 -4.870015 5.153672
C 2.101757 -3.577575 7.260230
H 3.964271 -4.653860 7.585152
C 1.201080 -3.017246 6.343559
H 1.886150 -3.504156 8.334846
H 0.286521 -2.511128 6.676634
C 3.727433 -4.383294 2.334040
H 4.212030 -5.171992 2.930533
H 4.489168 -3.647682 2.022371
H 3.273614 -4.848370 1.447680
C -0.356314 -1.910782 3.831866
H -0.393761 -1.320084 4.760735
H -1.234019 -2.579752 3.795763
H -0.368307 -1.212918 2.974990
C 1.599850 -3.061896 2.734851

TS [$MgCl_2$(THF)(DVL)BuOH]...NHO → [$MgCl_2$(THF)(DVL)BuO]$^-$...NHOH$^+$
76
geomtry on BP-86/def2-SVPD level incl. COSMO (ε = 7.58)
O -2.125956 2.325743 -0.425181
C -2.240255 2.660804 -1.827910
C -3.736439 2.942086 -2.038944
H -1.832757 1.809906 -2.396351
H -1.628697 3.567920 -2.028033
C -4.235486 3.393066 -0.634184
H -4.259366 2.024534 -2.364500
H -3.895225 3.714051 -2.813436
C -2.995672 3.250030 0.270176
H -5.055416 2.743093 -0.279244
H -4.607727 4.433502 -0.634947
H -3.195676 2.819420 1.264913
H -2.466207 4.221103 0.383269
Cl -0.531816 -0.289755 -1.477334
Cl -2.469965 0.364013 2.184847
Mg -0.738022 0.934726 0.595447
C 1.247580 4.887308 1.250605
C 2.385460 5.612685 0.520380
C 2.139644 5.539848 -0.989265
H 3.357790 5.138602 0.764656
H 2.443025 6.659673 0.868884
C 2.086547 4.088228 -1.430275

H	2.948662	6.037094	-1.557356
H	1.193000	6.054600	-1.251787
O	1.170415	3.270261	-0.620933
H	3.076133	3.600142	-1.347227
H	1.719825	3.964263	-2.462859
C	0.822181	3.579370	0.626122
H	0.331385	5.515821	1.257545
H	1.472293	4.684510	2.313715
O	0.107189	2.773598	1.238248
O	0.818178	-0.040237	1.499552
C	1.660508	0.442757	2.530446
H	0.906842	-1.273290	1.514089
C	3.013740	0.948851	2.016426
H	1.849513	-0.374948	3.265301
H	1.146250	1.251338	3.094182
C	3.933364	1.455806	3.135661
H	2.837513	1.756542	1.275983
H	3.511789	0.129669	1.457143
C	5.273088	1.999555	2.625340
H	4.118429	0.631210	3.856100
H	3.406794	2.246595	3.711096
H	5.832720	1.223168	2.066769
H	5.914393	2.345694	3.458449
H	5.120253	2.857046	1.939814
C	1.095865	-2.664228	1.425000
C	2.110347	-2.678473	0.276882
H	3.089537	-2.251544	0.557231
H	1.702808	-2.041531	-0.531106
H	2.274803	-3.684955	-0.161793
C	-0.266809	-3.199862	0.950145
H	-0.663437	-2.546668	0.150160
H	-1.030578	-3.263214	1.741653
H	-0.149537	-4.220413	0.525777
N	2.730963	-3.531683	3.186579
N	0.733113	-2.862288	3.892282
C	2.742430	-3.616756	4.580185
C	1.461924	-3.212669	5.024243
C	3.734425	-4.025645	5.481509
C	3.402099	-4.020828	6.850377
H	4.732670	-4.332507	5.143726
C	2.124669	-3.617822	7.293362
H	4.155455	-4.336917	7.585167
C	1.128928	-3.207319	6.386248
H	1.898085	-3.625615	8.368515
H	0.134005	-2.903436	6.735829
C	3.815279	-4.075188	2.374730
H	4.303240	-4.881283	2.948525
H	4.571134	-3.306297	2.130977

H 3.415533 -4.508182 1.448415
C -0.563295 -2.199398 3.961395
H -0.618923 -1.641220 4.911261
H -1.393460 -2.929195 3.931539
H -0.679262 -1.481656 3.132432
C 1.508588 -3.039084 2.752242

Details to the calculations of the metal-free deprotonation

For the metal-free deprotonation step, two minimum structures but no transition state corresponding to the proton transfer could be found. Instead, we optimized the reaction path using the NEB method [i-ii].

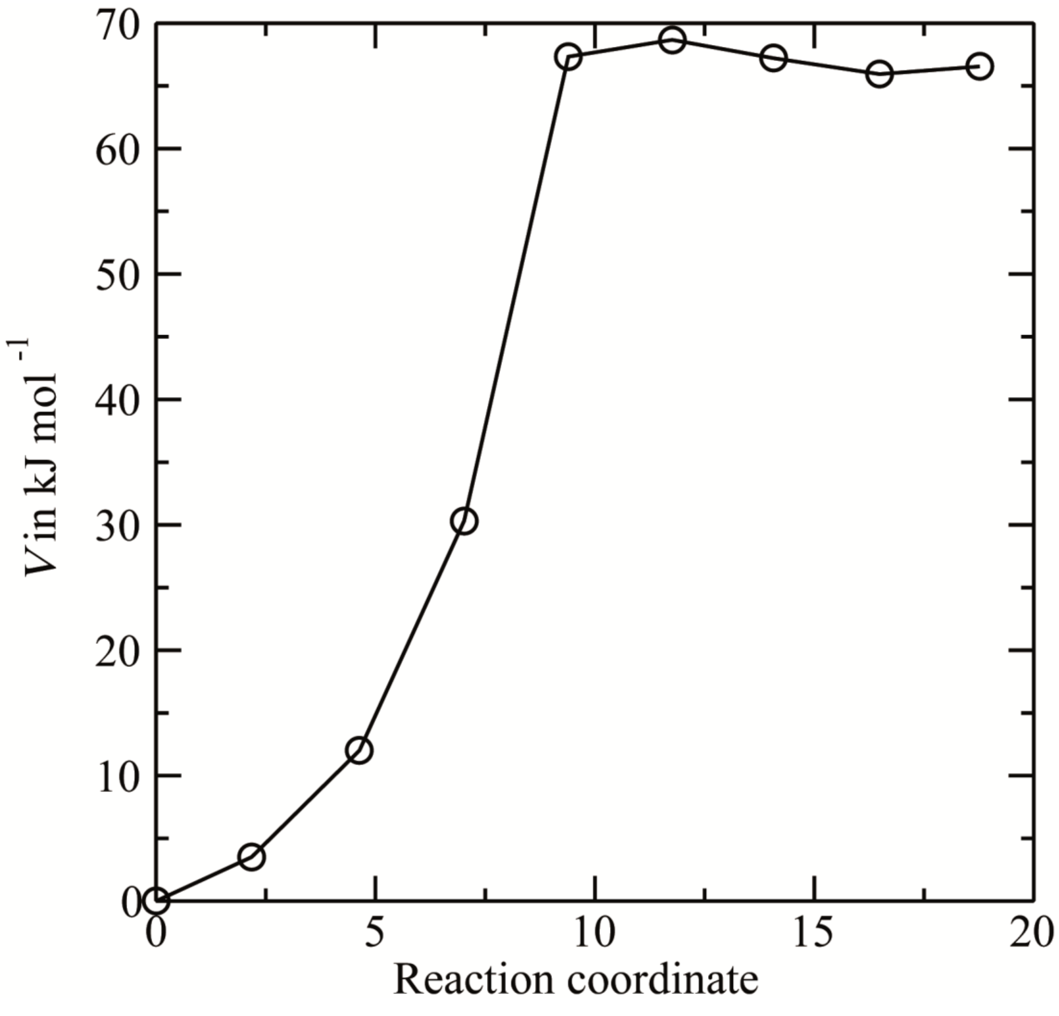

Figure S1. *Proton transfer in the absence of $MgCl_2$ (only NHO* ***7****, VL, THF, butanol present).*

References

[i] Henkelman, G.; Uberuaga, B. P.; Jónsson, H. A climbing image nudged elastic band method for finding saddle points and minimum energy paths. *J. Chem. Phys.* **2000**, *113*, 9901–9904.

[ii] Henkelman, G.; Jónsson, H. Improved tangent estimate in the nudged elastic band method for finding minimum energy paths and saddle points. *J. Chem. Phys.* **2000**, *113*, 9978–9985.
